# Low-Dimensional Semiconducting Silver (Germanium, Tin) Polyphosphides – Incommensurately Modulated Derivates of the HgPbP_14_ Structure Type

**DOI:** 10.1021/acs.inorgchem.5c03307

**Published:** 2025-09-10

**Authors:** Kathrin Vosseler, Aylin Koldemir, Rainer Pöttgen, Thomas Doert, Tom Nilges

**Affiliations:** † Synthesis and Characterization of Innovative Materials, TUM School of Natural Sciences, Department of Chemistry, Technical University of Munich, Lichtenbergstraße 4, Garching b. München 85748, Germany; ‡ Institut für Anorganische und Analytische Chemie, 271671 Universität Münster, Corrensstraße 30, Münster 48149, Germany; § Fakultät Chemie und Lebensmittelchemie, 9169 Technische Universität Dresden, Dresden 01062, Germany

## Abstract

Semiconductors with one-dimensional (1D) substructures are promising for next-generation optical and electronic devices due to their directional transport and flexibility. Representatives of this class include HgPbP_14_-type materials. This study investigates the related semiconductors Ag_1.7(1)_Ge_1.0(1)_P_14_ and Ag_1.4(1)_Sn_1.0(1)_P_14_. Single-crystal X-ray diffraction indicates that their structure is unconventional due to its incommensurate modulation. Both compounds crystallize orthorhombically in the (3 + 1)­D superspace group *Pnma*(0*β*0)*s*00 (No. 62.1.9.4). Ag_1.7(1)_Ge_1.0(1)_P_14_ (refined composition Ag_2.2(1)_Ge_1.3(1)_P_18.7(1)_) with the cell parameters *a* = 12.986(1) Å, *b* = 3.2648(4) Å, *c* = 10.841(1) Å, and a modulation wave vector *q* = (0, 0.39(1), 0), and Ag_1.4(1)_Sn_1.0(1)_P_14_ (refined as Ag_1.9(1)_Sn_1.3(1)_P_18.7(1)_) with *a* = 13.014(1) Å, *b* = 3.2602(4) Å, *c* = 10.905(1) Å, and *q* = (0, 0.42(1), 0) were investigated. Three structural models were generated, differing in modulation functions, site occupancies, and the split of one atomic position. Depending on the occupancy, the structure can be derived from Cu_2_P_20_, AgP_15_, or HgPbP_14_ -type materials. ^119^Sn Mössbauer spectroscopy confirms the +II oxidation state of tin in Ag_1.4(1)_Sn_1.0(1)_P_14_. Additional characterization was performed by scanning electron microscopy with energy-dispersive X-ray spectroscopy, X-ray photoelectron spectroscopy, angle-dependent Raman spectroscopy, and photoluminescence measurements. Single-crystal conductivity measurements revealed semiconducting behavior of Ag_1.7(1)_Ge_1.0(1)_P_14_ (0.2 S/cm).

## Graphical abstract

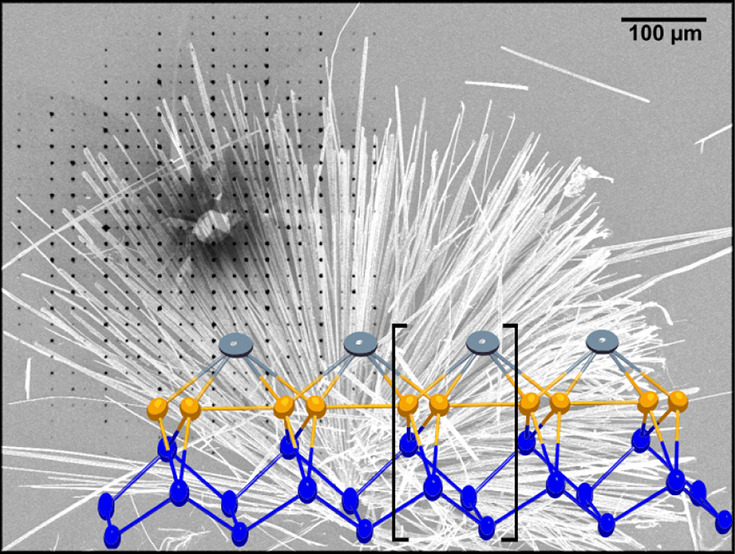

## Introduction

Semiconducting materials with one-dimensional substructures are interesting for novel optical and electronic devices due to their flexibility and directional charge transport.^1–3^ In the past, they were already used in sensors, photodetectors, photovoltaics, and energy storage applications.^4–6^

Selected quasi-1D semiconducting substructures are of interest in nonlinear optics as reported for GaSI.^7^ In this compound, an arrangement of corner-sharing GaS_3_I quasi-tetrahedra builds a helical motif. Furthermore, the material is easily exfoliable and possesses a band gap of 3.69 eV. The related materials, InSeI and GaSeI, show smaller band gaps of 2.45 and 2.85 eV, respectively, at room temperature.^8,9^

Polyphosphides are primarily interesting as they adopt manifold structures with various physical properties and chemical compositions.^10^ Not only in bare inorganic but also in metal organic chemistry, polyphosphide units are common structural elements in catalysts that trigger multiple properties.^11,12^ Phosphorus tends to form strong bonds with soft metals and acts as a donor atom in metal organic materials. Even low-dimensional aromatic entities are reported, for instance, for the [P_4_]^2–^ anion in Cs_2_P_4_·2NH_3._ ^13,14^

Anisotropic polyphosphide semiconductors, such as SnIP, SnBrP, AgP_15_, or Cu_2_P_20_, were investigated in our group in order to find new quasi-1D or -2D materials that can, e.g., form 1D-2D heterosubstructures.^15–18^ SnXP (with X = I, Br) are quasi-1D semiconductors that are characterized by a double-helical arrangement of [Sn–I] and [P]-helices.

AgP_15_ and Cu_2_P_20_ are characterized by tubular polyphosphide subunits coordinated by Ag or Cu cations, both comprising a layered arrangement of those tubular subunits. All four examples show van der Waals-type interactions between the neutral structure fragments that allow delamination to nanosized crystals. Such delaminated materials can be applied to form hybrids suitable for optoelectronic applications like water splitting. As a first example, Ott and Reiter et al. described SnIP and halide-doped carbon nitride in 2019, featuring an almost 4-fold increase in the water-splitting performance compared with the bare materials.^19,20^

In this study, we focus on materials of the HgPbP_14_ structure type, another representative of a quasi-1D material. The quasi-1D behavior results from its strongly anisotropic crystal structure and the van der Waals interactions between neighboring structural units, mostly originating from the lone-pair interactions of the polyphosphide subunit. Concerning its elemental composition, this compound is rather unusual in phosphorus chemistry. Hg does not form a binary phase with phosphorus, and for Pb, only one representative, namely PbP_7_, is known.^21^ PbP_7_ can be regarded as a derivative of the black phosphorus structure. The phosphorus substructure in PbP_7_ consists of P_6_ rings in chair conformation, which exhibit trans-edge condensation, as is the case in black phosphorus. In contrast to that structure, the P_6_ rings are further connected by a phosphorus atom, forming a three-dimensional network.^21^ HgPbP_14_ combines the two rare cases of almost nonreactive elements with phosphorus. This study investigates Ag_1.7(1)_Ge_1.0(1)_P_14_, and Ag_1.4(1)_Sn_1.0(1)_P_14_, that are related to the HgPbP_14_ structure type.

## Materials and Methods

### Materials

#### Preparation of Ag_1.7(1)_Ge_1.0(1)_P_14_, Ag_1.4(1)_Sn_1.0(1)_P_14_

The compounds were synthesized in a chemical vapor transport reaction from a molar 1:1:14 mixture of silver (AlfaAesar, shot, 99.999%), germanium (Chempur, pieces, 99.999%), or tin (Chempur, pieces, 99.9999%), and red phosphorus (Chempur, pieces, 99.9999%) in an overall amount of 250 mg (phosphorus excess). The phosphorus pieces were ground under a protective atmosphere in a glovebox prior to usage. After transfer of the phosphorus into the reaction ampules the remaining starting materials were added under the same protective gas conditions. All other starting materials were stored under ambient conditions prior to usage. After the addition of 15 mg germanium­(IV) iodide (Thermo Scientific, powder, 99.999%) or 15 mg tin­(IV) iodide, respectively, the starting materials are sealed into evacuated silica glass ampules of 0.8 cm inner diameter and a wall thickness of 0.1 cm. The mixture was kept in a muffle furnace for 7 days at 823 K. They were subsequently cooled to room temperature. Crystals of both compounds were formed as needle bundles at the ampule walls separated from the bulk residue or directly on the bulk phase. The bulk consists of binary and ternary side phases. Single crystals are obtained by prolonging the cooling process. A direct synthesis of the title compounds from stoichiometric amounts of the elements—without the utilization of chemical transport—did not lead to a phase pure material. White phosphorus tends to be finely dispersed over the entire ampule, leading to a high probability of self-ignition after application to air (Caution). The same is true if a transport reaction is applied and one deviates from the herein given synthesis protocol. Attention is also required, if the total amount of starting materials is increased, the vapor pressure of the starting materials (especially red phosphorus) prior to the formation reaction will become critical, and disintegration of the ampule may occur.

### Characterization Methods

Powder X-ray diffractograms were recorded with a STOE STADI P powder diffractometer using Cu Kα_1_ radiation (λ = 1.54060 Å, curved Ge(111) monochromator), which is equipped with a position-sensitive Dectris Mythen 1K detector. The data were collected between 5 and 79° or 5 and 105° 2θ using a step width of 0.015°. Data analysis was conducted using the STOE WinXpow software package.^22^

The single-crystal XRD data were collected on a STOE STADIVARI diffractometer equipped with a Dectris PILATUS 300 K hybrid pixel detector at 300(1) K using an Oxford Cryostream plus system using Mo K_α1/2_ radiation (λ = 0.71073 Å) and on a Bruker Apex II diffractometer at room temperature using Mo K_α1/2_ radiation (λ = 0.71073 Å). A numerical absorption correction was applied.

The structure was solved using the charge-flipping function implemented in the Jana2006 program suite.^23^ We were able to observe first- and second-order satellite reflections. However, due to the poor intensities of the second order satellites, both structures were refined using first-order satellites only.

Secondary electron microscopy (SEM) and semiquantitative energy-dispersive X-ray spectroscopy (EDS) were performed on a JEOL-IT 200 equipped with a JEOL JED-2300 EDS detector.

The ^119^Sn Mössbauer spectroscopic experiment on Ag_1.4(1)_Sn_1.0(1)_P_14_ was conducted with a Ca^119m^SnO_3_ source in the usual transmission geometry. A palladium foil of 0.05 mm thickness was applied to reduce the tin K X-rays emitted by this source. The sample (about 35 mg) was placed in a thin-walled PMMA container with a diameter of 15 mm. The temperature of the absorber was set to 78 K using a commercial liquid nitrogen-bath cryostat, while the source was kept at room temperature. Fitting of the experimental data was done with the WinNormos for the Igor7 program package^24^ and graphical editing with the program CorelDRAW2017.^25^

Raman spectroscopy was performed using a Nd:YAG laser with an excitation wavelength of 532.124 nm (*P*_Laser_ = 0.794 mW). The center wavelength was adjusted to 549.070 nm and the spectral center to 580 rel. 1/cm. This was coupled to a confocal microscope (WITec Alpha 300 R) with a 100× (0.9 NA) objective, a 600 g/mm, BLZ = 500 nm grating, and an Oxford UHTS300S_VIS Throughput spectrometer.

X-ray photoelectron spectroscopy (XPS) spectra were obtained with a Kratos Axis Supra spectrometer using monochromatic Al Kα radiation (*hν* = 1486.6 eV) and a total power of 225 W under ultrahigh vacuum (<1.33 × 10^–7^ Pa). The survey scans were conducted between 0 and 1200 eV BE in two sweeps using 100 ms dwell time and a step size of 0.5 eV. All detailed scans were measured using a step size of 0.1 eV. For C 1s, a dwell time of 200 ms and three sweeps were used. Ag 3d was measured using 2500 ms dwell time and five sweeps. The P 2p region was assessed using 830 ms dwell time and five sweeps. The binding energy values were calibrated using the C 1s photoemission peak for adventitious hydrocarbons at 284.8 eV.

Photoluminescence measurements were also performed using an Nd:YAG laser with an excitation wavelength of 532.124 nm (*P*_Laser_ = 0.5 mW). The center wavelength and the spectral center were adjusted to 660 nm. This was coupled to a confocal microscope (WITec Alpha 300 R) with a 100× (0.9 NA) objective and a 300 g/mm, BLZ = 500 nm grating, and an Oxford UHTS300S_VIS Throughput spectrometer.

The resistivity measurement was conducted in vacuum (∼1 × 10^–6^ mbar) using a Yokogawa GS200 voltage source and an Agilent 34410A current meter with an Ithaco 1211 as an upstream I–V amplifier.

## Results and Discussion

To understand the modulation and complex structure of the title compounds it is useful to first describe the structural chemistry of the HgPbP_14_ type adopted by compounds with the general sum formula **M1M2**P_14_. Here, **M1** is a group 11 or 12 transition metal cation, and **M2** is the lone pair cation Sn, Pb, or Sb. In 1955, HgPbP_14_ was reported for the first time by Krebs et al.^26^ Its ionic description to illustrate the bonding situation is (M1)^2+^[(M2)^2+^(P^0^)_10_(P^1–^)_4_]^2–^ where **M1** = Hg, Zn, Cd and **M2** = Pb, Sn.^26–30^ This structure type is related to the structure of fibrous phosphorus s, which consists of interconnected [P21] repetition units (see below, Figure 4). The [P21] units can be described as $\overset{1}{\infty }$ ­([P8]­P2­[P9]­P2­[) strands according to the Baudler nomenclature^31–34^ which are interconnected via the [P9] unit.^35^ In contrast, the HgPbP_14_ structure is built of $\overset{1}{\infty }$ ­(]­P2­[P2**M2**]­P2­[P3]­P2­[P3])^2–^ strands. The transition metal **M1** is connected to the [P3] building blocks, forming a [P8**M1**] cage. The **M1** cation is distorted tetrahedrally coordinated by phosphorus atoms interlinking the individual strands. Materials with the HgPbP_14_ structure type show semiconducting properties with band gaps in the range of 0.4 ± 0.2 eV for HgPbP_14_ up to 1.6 ± 0.1 eV for ZnSnP_14_.^28^

The $\overset{1}{\infty }$ ­(]­P2­[P2**M2**]­P2­[P3]­P2­[P3])^2–^ anion itself is rather robust and also allows other than 2+ charged cations to be coordinated on both cation sites. Examples are the compounds Au_0.64_Sn_1.36_P_14_ and Cu_0.73_Sn_1.27_P_14_, where the **M1** position is occupied with Au^+^ /Cu^+^ and Sn^2+^ while the **M2** position is unaffected (M^2+^).^29,30^ The gold compound was initially interpreted according to the Zintl–Klemm concept as [(Au^+^)_0.64_(Sn^4+^)_0.36_]^2+^[Sn^2+^P_14_]^2–^, assuming tin in two oxidation states.^29^ Subsequent analysis by Mössbauer spectroscopy showed the absence of Sn^4+^ leading to the description [(Cu^+^)^1–*x*^(Sn^2+^)_*x*_]^(1+*x*)+^[(Sn^2+^)­(P_14_)]^(1+*x*)–^.^30^ Due to the enlargement of the *b*-axis, the introduction of a split atom position becomes necessary in those compounds. It can also be observed in phosphorus-containing clathrates like Sn_24_P_19.3_I_8_ and Sn_24_As_19.3_I_8_.^36^

The structural flexibility of this material class is even increased by realizing *M* ^3+^ cations, like Sb^3+^, on the **M2** position. To reach charge neutrality and an overall charge of +IV, the **M1** position is now occupied by an *M* ^+^ cation like Ag^+^.^37^ EDS measurements of AgSbP_14_ showed an almost equimolar ratio of Ag^+^ and Sb^3+^, and ^121^Sb Mössbauer confirmed the +III oxidation state of antimony.^30,37^

If we now continue to combine an *M* ^+^ cation (other than Au or Cu) on the **M1** site and offer a second element like Ge or Sn capable to adopt either the *M* ^2+^ or *M* ^4+^ oxidation state on the **M2** site, the question arises as to whether this can also lead to a HgPbP_14_ type material. To answer this question, we started to investigate the Ag–Ge–P and Ag–Sn–P systems. Only a few ternary compounds in the Ag–Ge–P and Ag–Sn–P systems are known to date. Ternary phase diagrams are shown in Figure 1. The two components Ag and Ge are not mixable; therefore, no discrete binary phases are stated in the ternary phase diagram a).^38^

**Figure 1:**
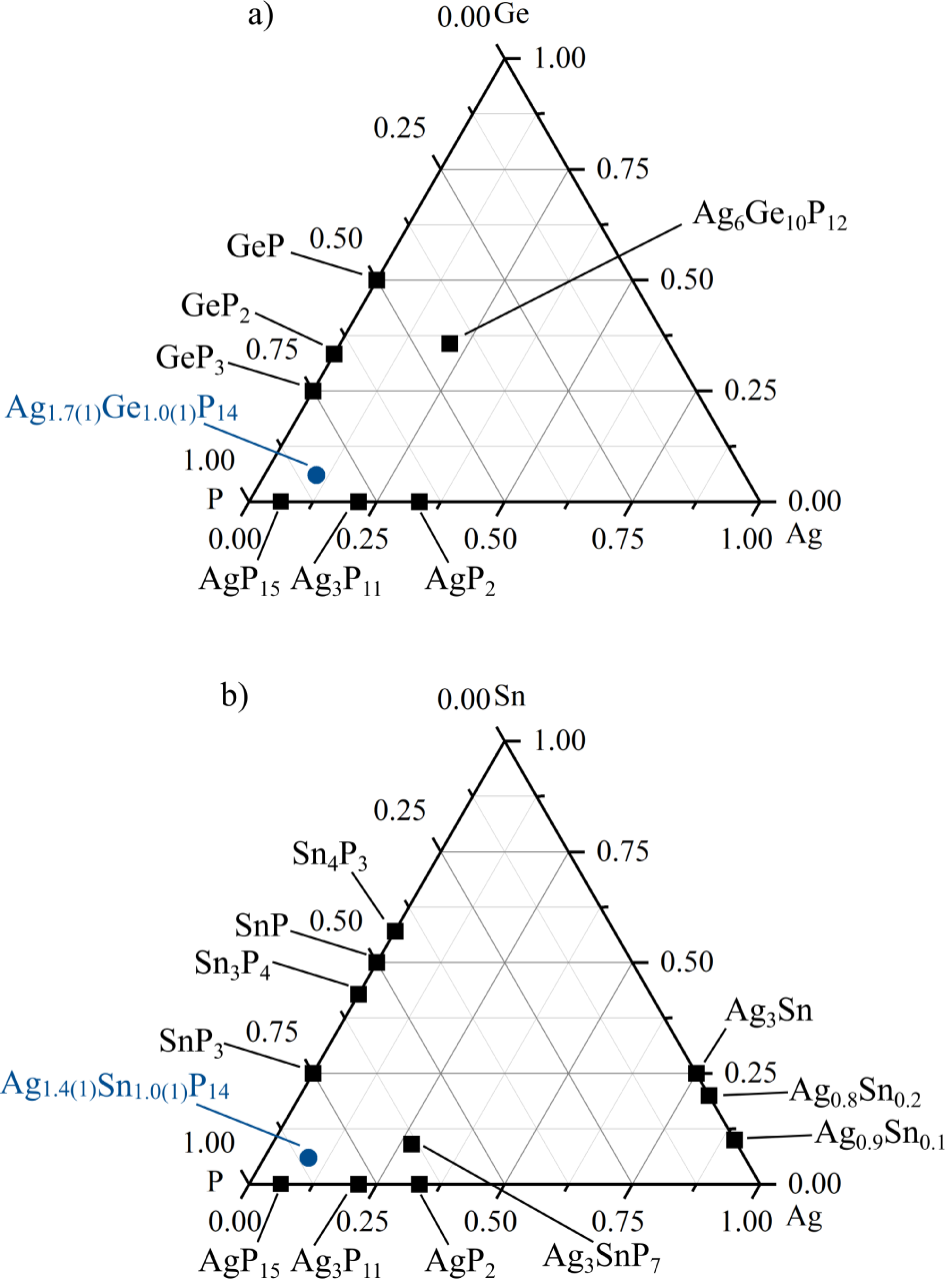
(a) Ag–Ge–P phase diagram: the black squares show all binary phases of Ag–P and Ge–P, as well as Ag_6_Ge_10_P_12_,^39^ the blue circle shows the title compound Ag_1.7(1)_Ge_1.0(1)_P_14_. (b) Ag–Sn–P phase diagram: the black squares show all binary phases of Ag–P, Sn–P, and Ag–Sn as well as Ag_3_SnP_7_, and the blue circle shows Ag_1.4(1)_Sn_1.0(1)_P_14_. Citations concerning the binary and known ternary compounds are given in the electronic Supporting Information.

As is evident in the phase diagrams above, only a few silver-containing ternary phases exist in the Ag–Ge–P and Ag–Sn–P systems. The ternary compound Ag_6_Ge_10_P_12_ is an air-stable phosphide that exhibits thermoelectric properties. Its crystal structure is related to the tetrahedrite (Cu_12_Sb_4_S_13_) structure type containing Ag_6_ clusters.^39,40^

The only other ternary phase in the Ag–Sn–P system is Ag_3_SnP_7_, a semiconductor with a band gap of approximately 0.2 eV. This polyphosphide is built by $\overset{1}{\infty }\left[P7\right]$ chains of interconnected six-membered phosphorus rings further connected by Ag_3_Sn heteroclusters.^41^ The bonding situation in this compound was further analyzed by ^119^Sn Mössbauer spectroscopy.^37^

A compound with the postulated composition “AgSnP_14_” has also been mentioned in the literature, but no crystal structure has been determined yet.^29,30^

Throughout the synthesis procedure described above, needle-shaped gray to black crystals could be obtained growing on top of a bulk phase as well as growing on the ampule walls. Figure 2 shows secondary electron­(SE)-SEM images of Ag_1.7(1)_Ge_1.0(1)_P_14_. From these images, we derived an average needle length of 392 μm and an average diameter of 4 μm. These values lead to an aspect ratio of ≈100, typical for fibers, nanowires, and nanotubes.^42^ The materials strongly tend to delaminate, as indicated by the red circle in Figure 2b.

**Figure 2:**
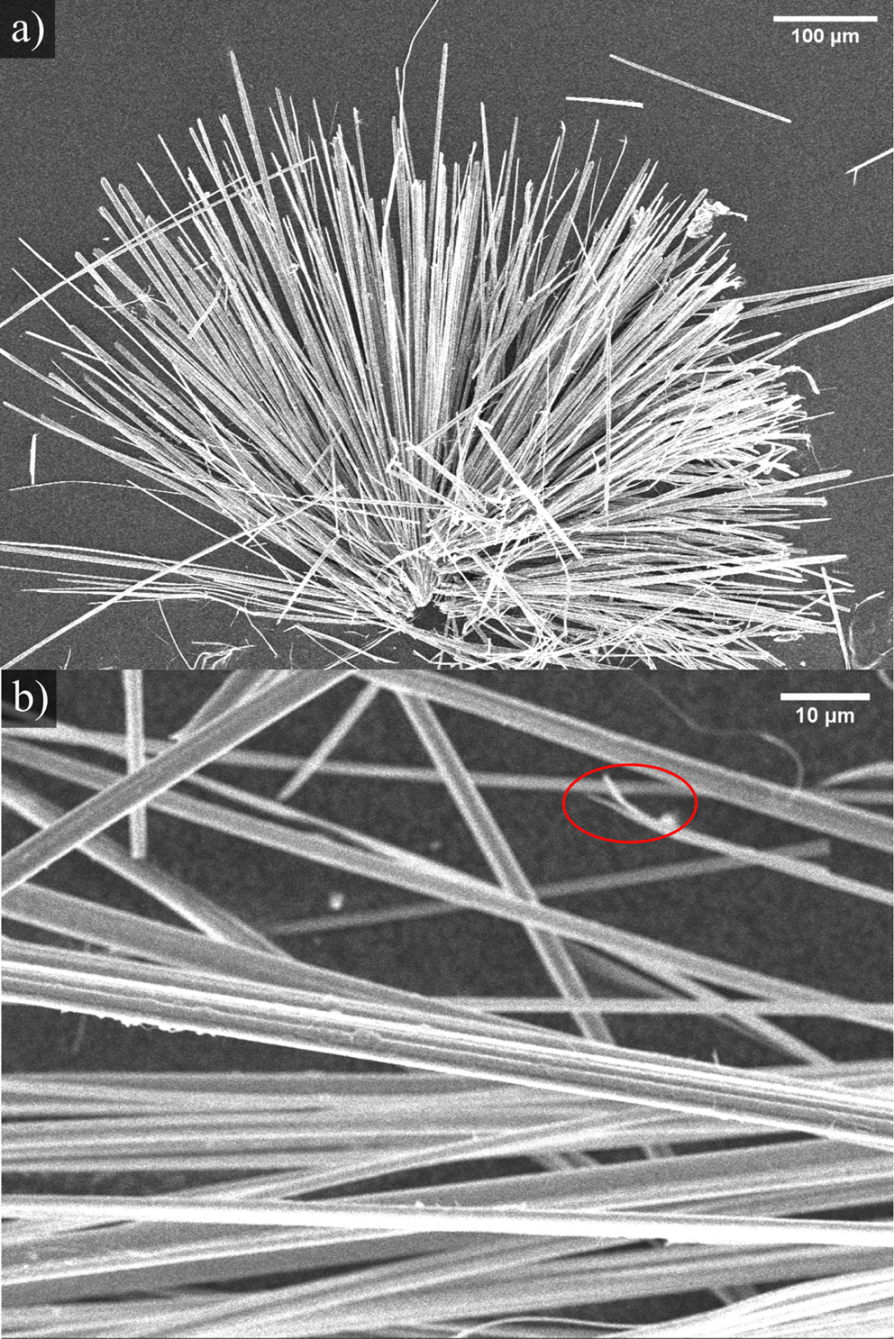
(a) SE-SEM overview image of Ag_1.7(1)_Ge_1.0(1)_P_14_. (b) Delamination tendency of Ag_1.7(1)_Ge_1.0(1)_P_14_ is illustrated by a red circle in the magnified image.

### Crystal Structure Determination and Discussion

X-ray single-crystal and powder diffraction were applied to determine the crystal structures of the title compounds. Derived from the symmetry and cell content, we calculated a composition of Ag_2.2(1)_Ge_1.3(1)_P_18.7(1)_/Ag_1.9(1)_Sn_1.3(1)_P_18.7(1)_ with *Z* = 1. To compare this to the closely related HgPbP_14_ structure type, we normalized the phosphorus content to 14, leading to sum formulæ of Ag_1.7(1)_Ge_1.0(1)_P_14_, and Ag_1.7(1)_Ge_1.2(1)_P_14_, respectively. In the following, we will use the HgPbP_14_-like notation to illustrate the close relation to this structure type. An analysis of the Ag_1.7(1)_Ge_1.0(1)_P_14_ single-crystal intensity data at 300 K and structure solution using the Superflip Routine^43^ in Jana 2006 and Jana2020,^23,44^ led to an orthorhombic structure model adopting the (3 + 1)­D superspace group *Pnma*(0*β*0)*s*00 (Nr. 62.1.9.4), with the lattice parameters *a* = 12.986(1) Å, *b* = 3.2648(4) Å, *c* = 10.841(1) Å and the modulation wave vector **q** = (0, 0.39(1), 0). Figure 3 shows the intensity summation of the (0*kl*) to (4*kl*) sections of reciprocal space. This summation was conducted to increase the intensity and, hence, the visibility of the satellite reflections. As a result, up to second-order satellite reflections are visible, clearly showing the incommensurate nature of the crystal (Figure 3a).

**Figure 3:**
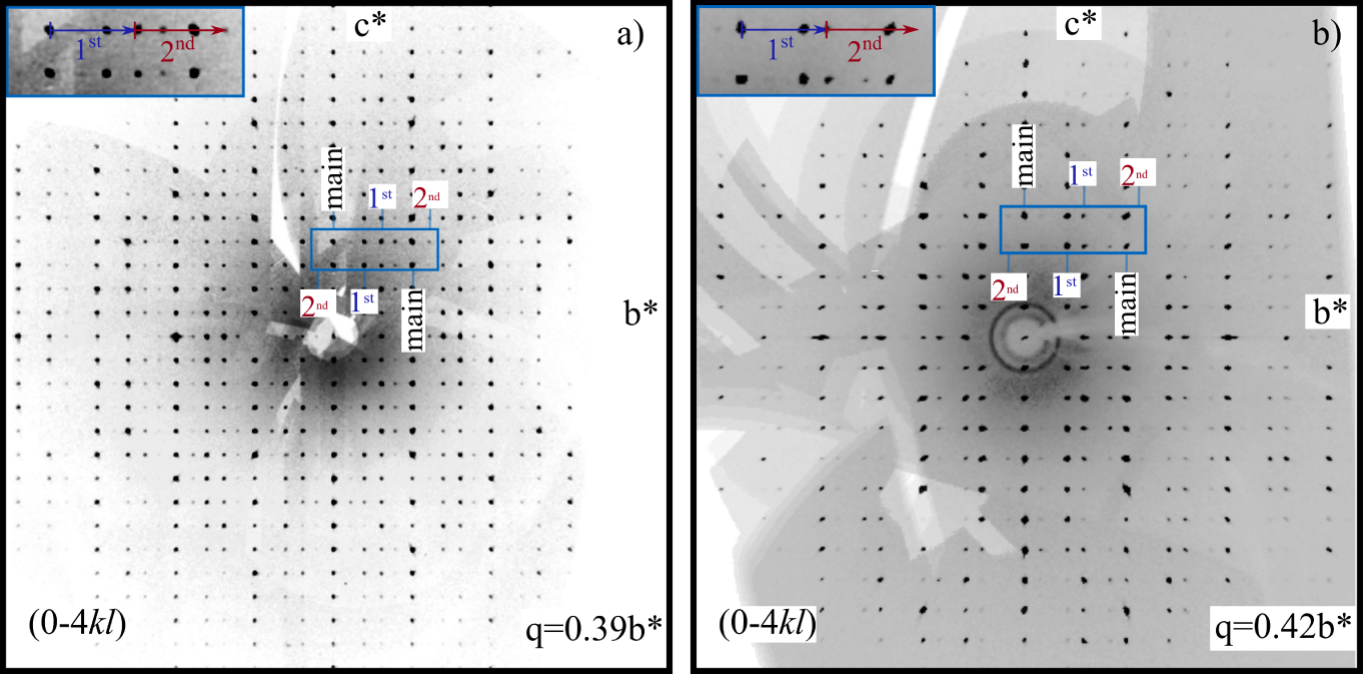
(a) Summation of the five sections (0*kl*) to (4*kl*) reconstructed reciprocal space of Ag_1.7(1)_Ge_1.0(1)_P_14_. Inset: The dark blue arrow represents the set of projected *h*031 first-order satellites of the set of *h1*30 main reflections. The red arrow indicates a set of projected second-order *h*1̅32 satellite reflections. (b) Summation of the intensities of the five reconstructed reciprocal space sections (0*kl*) to (4*kl*) of Ag_1.4(1)_Sn_1.0(1)_P_14_. Inset: The dark blue arrow represents the set of projected first-order satellites *h*031 of the set of *h1*30 main reflections. The red arrow indicates a set of projected second-order *h*1̅32 satellite reflections.

In addition, the crystal structure of silver tin polyphosphide was determined, as it is supposed to be isostructural to the previously mentioned silver germanium polyphosphide. Analysis of the single-crystal intensity data resulted in a unit cell with cell parameters *a* = 13.014(1) Å, *b* = 3.2602(4) Å, *c* = 10.905(1) Å, a sum formula of Ag_1.9(1)_Sn_1.3(1)_P_18.7(1)_, and *Z* = 1. It crystallizes orthorhombically, in superspace group *Pnma*(0*β*0)*s*00 (Nr. 62.1.9.4), with the modulation wave vector **q** = (0, 0.42(1), 0). Besides strong first-order satellites, we could observe weaker second-order satellite reflections (Figure 3b); a summation of the (0*kl*) to (4*kl*) lattice planes was done again to increase the intensity of the satellites for better visualization.

The atomic displacement parameters were refined anisotropically in most cases, dependent on the applied structure model. Sections of Fourier maps (*F*_0_) around the atom positions (Figures S1–S3, S5, and S6), so-called de Wolff sections, indicate that for all atom positions, positional, and in some cases, occupational and ADP modulation waves have to be used to describe the electron density properly. Full-matrix least-squares refinements are based on *F* ^2^. Table 1 and the supplement data section provide additional crystallographic information and refinement details.

**Table 1:** Single-Crystal XRD Data of Ag_1.7(1)_Ge_1.0(1)_P_14_ and Ag_1.4(1)_Sn_1.0(1)_P_14_, Measured at Room Temperature

	Ag_1.7(1)_Ge_1.0(1)_ P_14_	Ag_1.4(1)_Sn_1.0(1)_P_14_
Refined composition	Ag_2.2(1)_Ge_1.3(1)_P_18.7(1)_	Ag_1.9(1)_Sn_1.3(1)_P_18.7(1)_
Molar mass (g mol^–1^)	916.8	935.1
Modulation model	“Split atom model”	“Split atom model”
Crystal shape/color	Needle/black	
Crystal system	Orthorhombic	
Super space group	*Pnma*(0*β*0)*s*00	
*Z* (per unit cell)	1	1
*a* (Å)	12.9856(14)	13.0139(14)
*b* (Å)	3.2648(4)	3.2602(4)
*c* (Å)	10.8410(12)	10.9053(12)
*V* (Å^3^)	459.61(9)	462.69(9)
*q* vector	0.39	0.42
ρ_calc._ (g cm^–3^)	3.32	3.37
Diffractometer	Bruker Apex II	STOE StadiVari
Radiation (Å)	0.71073 (Mo K_α1/2_)	
μ (cm^–1^)	6.4	5.3
*F*(000)	429	435
θ range (°)	2.33–32.6	2.12–44.82
hkl range	–19/+19, – 5/+5, – 16/+16	–19/+19, −5/+5, −16/+15
No. of reflections	31918	37585
*R*_int_	0.166	0.128
Data/parameters	1678/76	1686/98
R/wR [I > 3σ (I)] (all)	0.0305/0.0648	0.0490/0.1006
R/wR [all] (all)	0.0555/0.0692	0.1066/0.1167
R/wR [I > 3σ (I)] (main)	0.0233/0.0538	0.0302/0.0604
R/wR [all] (main)	0.0320/0.0555	0.0530/0.0669
R/wR [I > 3σ (I)] (satellites)	0.0430/0.0792	0.0849/0.1701
R/wR [all] (satellites)	0.0923/0.0867	0.1822/0.1990
Goodness of fit	1.59	1.11
Res. elec. dens. max/min (e Å^–3^)	–0.80/+0.51	–1.75/+1.39

Ag_1.7(1)_Ge_1.0(1)_P_14_ and Ag_1.4(1)_Sn_1.0(1)_P_14_ consist of a polyphosphide substructure that can be derived from the fibrous phosphorus structure. Figure 4 denotes the structure relation between fibrous red phosphorus, Cu_2_P_20_, AgP_15_, and the [P14] substructure in the HgPbP_14_-structure type.

**Figure 4:**
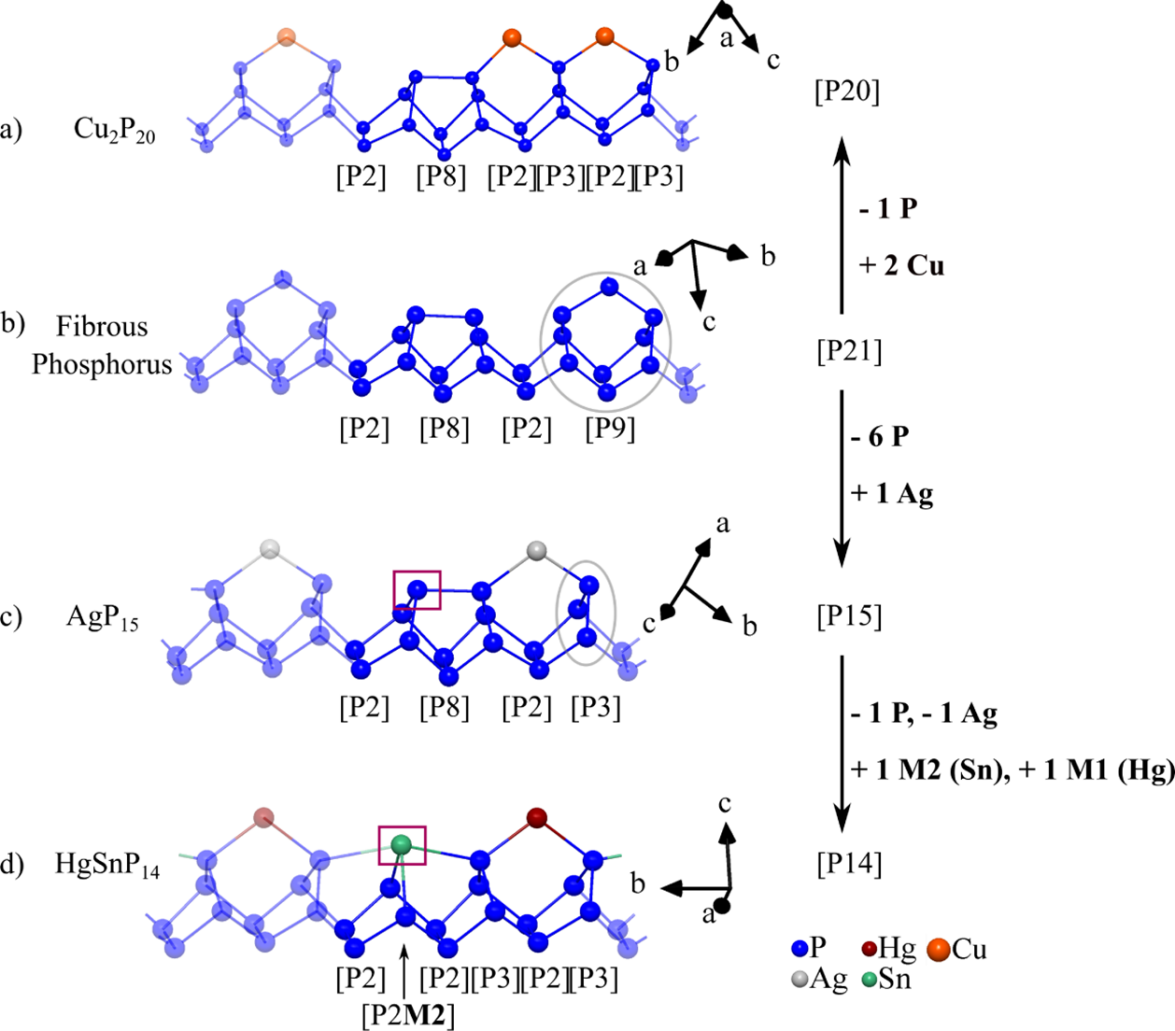
(a) Illustration of a single phosphorus strand in Cu_2_P_20_,^18^ quasi-molecular subunits illustrate the [P20] unit. (b) Single strand in fibrous phosphorus,^35^ quasi-molecular subunits illustrate the [P21] unit. (c) Representation of a single strand in AgP_15_, the [P3] unit is marked by a gray circle and one P atom by a violet box.^17^ (d) Illustration of a single strand in the HgSnP_14_.^28^ All cell axes are given. Dark blue atoms represent independent P atoms in fibrous phosphorus (21 atoms), Cu_2_P_20_ (20 atoms), AgP_15_ (15 atoms), and HgSnP_14_ (14 atoms). The gray circles and violet boxes illustrate the main differences between the three structures. A schematic scheme of the changes is given on the right-hand side. Phosphorus subunits are classified according to the Baudler nomenclature.

The fibrous phosphorus structure consists of $\overset{1}{\infty }$ ­([P8]­P2­[P9]­P2­[) units (Figure 4b) according to the Baudler nomenclature.^31–34^ If a [P9] unit of fibrous phosphorus is replaced by a [P3] unit (gray circle, Figure 4), a $\overset{1}{\infty }(\left[P8\right]P2\left[P3\right]P2\left[\right)$ unit results that carries a 1– charge. Silver now coordinates the strand, bridging fractions of the former [P8] cage and the [P3] unit (see Figure 4c). To evolve the structure further to the HgPbP_14_ structure type (Figure 4d), Hg replaces Ag on the **M1** site. One phosphorus atom of the [P8] cage in AgP_15_, which is marked by a violet box in Figure 4 center, is substituted by Sn creating a new [P2**M2**] moiety. Now, one ends up with the $\overset{1}{\infty }(\left[P2M2\right]P2\left[P3\right]P2\left[P3\right]P2\left[\right)$ polyphosphide moiety realized in the HgPbP_14_ structure type. Another important binary phase that can be derived from fibrous phosphorus is Cu_2_P_20_.^18^ Abstraction of the bridging P atom in fibrous phosphorus leads to the formation of a [P20]^2–^ polyphosphide substructure that can be described as a $\overset{1}{\infty }$ ­([P8]­P2­[P3]­P2­[P3]­P2­[) moiety (Figure 4a). The two resulting 2-bonded phosphorus atoms are coordinated by two Cu^+^ ions.

In all structure types discussed, the polyphosphide substructure shows a pentagonal cross-section that runs along a particular axis. However, the title compounds deviate from this idealized periodicity by adopting an incommensurately modulated structure composed of all three aforementioned polyphosphides.

In order to describe the incommensurate part of the modulated crystal structure best, we refined three different structure models. The three structure models differ primarily in the description of the **M2** position, while the **M1** position and the remaining phosphorus substructure exhibit minimal deviation from each other. In the first model, the **M2** cation lies directly on a mirror plane within the unit cell of the model in space group *Pnma*. It is further referred to as the “mirror plane model”. In the second and third structure models, the **M2** cation is moved slightly off the mirror plane, resulting in a split atom position. Furthermore, in the second model, the occupational modulation waves to describe the **M2** site are sinus/cosine functions, while in the third model, the occupational modulation is defined by crenel functions. The second model is from now on referred to as the “split atom model”, and the third one as the “crenel model”. A refinement for Ag_1.4(1)_Sn_1.0(1)_P_14_ using the “crenel model” led to negative displacement parameters on the **M2** position and was therefore not considered further. Note that refinements in the respective noncentrosymmetric superspace group *Pn*2_1_ *a*(0*β*0)*s*00 do not allow for a better structure fit but result in a large number of correlations, which lead to an unstable refinement.

In the following, we discuss the principal structure features and illustrate the differences of the aforementioned models in detail.

In all models, the phosphorus substructure is somewhat similar, and slight variations occur due to the different descriptions of the **M2** site. The entire phosphorus substructure shows a slight positional modulation dependent on the modulation applied on the **M2** site. Compared to compounds adopting the nonmodulated HgPbP_14_ structure type (*b*-axis ∼9.8 Å), the *b*-axis of the basic structure of the modulated title compounds is approximately three times smaller. As a result of this aspect, the phosphorus substructure is described by a [P2] unit and the [P2**M2**] entity per basic unit cell, creating the illustrated polyphosphide substructure as depicted in Figure 5. The bond distances within the phosphorus backbone are between 2.2 and 2.3 Å in all three structural models and thus agree with the typical bond distances of covalently bonded phosphorus. These lie between 2.17 and 2.30 Å, with shorter contacts down to 2.15 Å for higher bond orders and weak interactions extending up to 2.39 Å.^10^

**Figure 5:**
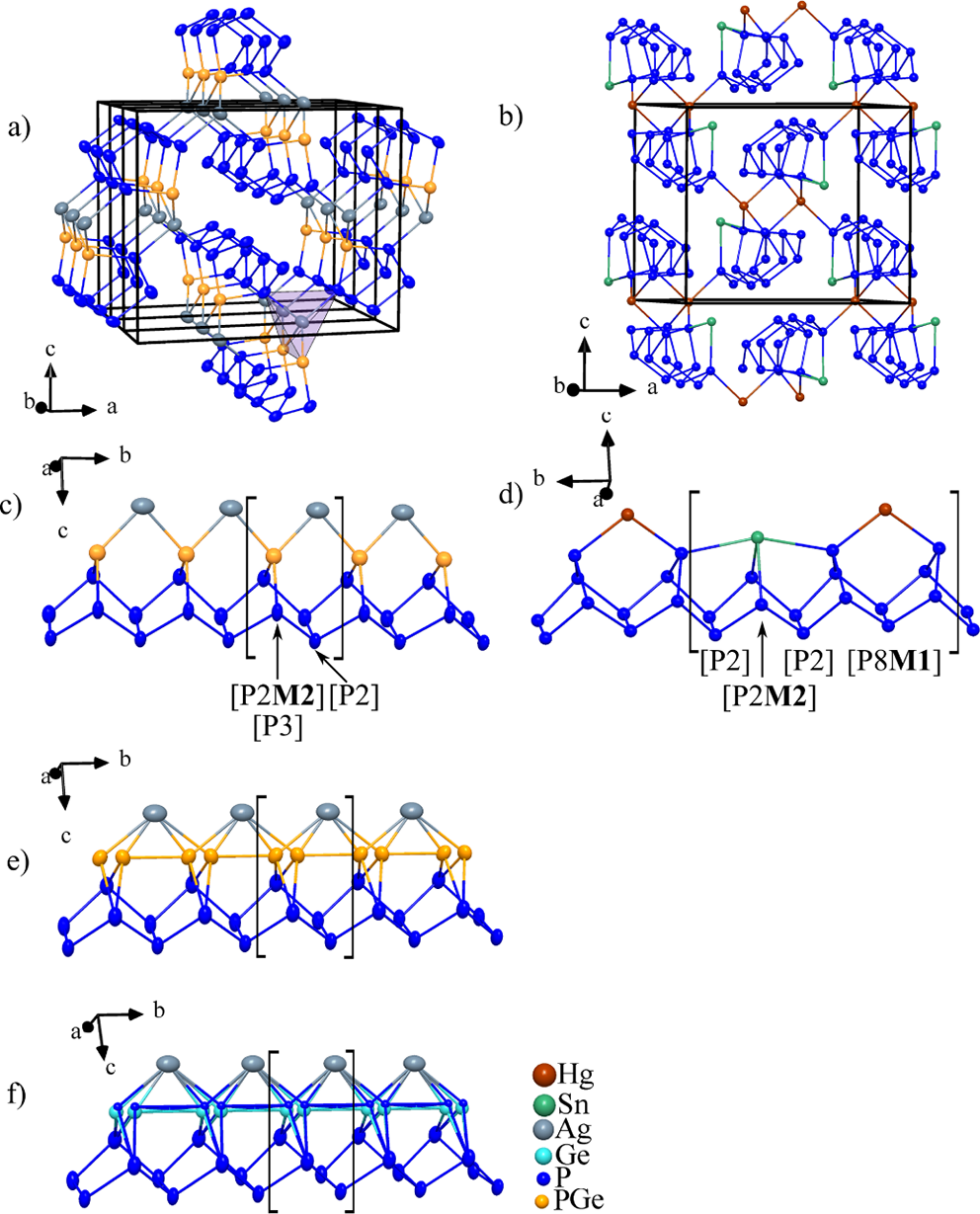
(a) Average structure of Ag_1.7(1)_Ge_1.0(1)_P_14_ using the “mirror plane model” with three unit cells drawn. (b) Unit cell of HgSnP_14_.^28^ c) View along the *a*-axis of Ag_1.7(1)_Ge_1.0(1)_P_14_ using the “mirror plane model”. (d) [P14] unit in HgSnP_14_.^28^ (e) View along the *a*-axis of the average structure of the “split atom model”. (f) View along the *a*-axis of the average structure of the “crenel model”. All cell axes are given, and the phosphorus subunit in HgSnP_14_ and the title compound using the “mirror plane model” is indicated. ADP parameters drawn at 90%.

The Ag^+^ cation on the **M1** site connects three independent parallel polyphosphide strands to a 3D arrangement. In all three models, the silver on the **M1** site needs to be described using occupational and displacive modulation waves. As a result, the **M1** site shows occupancy factors (s.o.f.s) of up to ∼0.5. Silver is coordinated distorted-tetrahedrally by phosphorus of the polyphosphide backbone and either phosphorus or germanium/tin on the **M2** site. The Ag–P bond distances to the phosphorus in the backbone are between 2.4 and 2.6 Å, slightly shorter than the Ag–P distances of 2.5–2.8 Å found in other (poly)­phosphides such as AgP_15_, AgP_2_, and Ag_3_P_11_.^17,45,46^

In the case of Ag_1.7(1)_Ge_1.0(1)_P_14_ and Ag_1.4(1)_Sn_1.0(1)_P_14_, a displacive and occupational modulation is needed to describe the situation on the **M2** site (Figure 5b). In the title compounds, a mixed occupancy of germanium/tin and phosphorus, with s.o.f’s of approximately 0.3 for germanium/tin and 0.7 for phosphorus, led to the best refinement results. This mixed occupancy by phosphorus and tin/germanium on the **M2** site affects the polyanion structure, as stated above. If phosphorus is present on the **M2** site (named P1 in the cif files), the structure adopts the AgP_15_ structure motif. However, if germanium or tin is present on the **M2** site, the compound agrees with the typical P_14_ moiety of the HgPbP_14_ structure type.

Figure 5 shows the average structure derived from the three structure models of Ag_1.7(1)_Ge_1.0(1)_P_14_ and their phosphorus substructures in relation to HgSnP_14_. To compare Ag_1.7(1)_Ge_1.0(1)_P_14_ with HgSnP_14_ in Figure 5, one has to take into account that the unit cell of Ag_1.7(1)_Ge_1.0(1)_P_14_ needs to be tripled in the crystallographic *b* direction.

Bond lengths that are defined by the occupancy and distances between the modulated sites are essential information to judge the quality of the three structure models. A maximum occupancy limit of 0.5 is applied to calculate interatomic distances. This limit is chosen to illustrate the predominantly occurring bonds in the structure. Figure 6 shows the resulting graphic representation of the distribution of the bond lengths.

**Figure 6:**
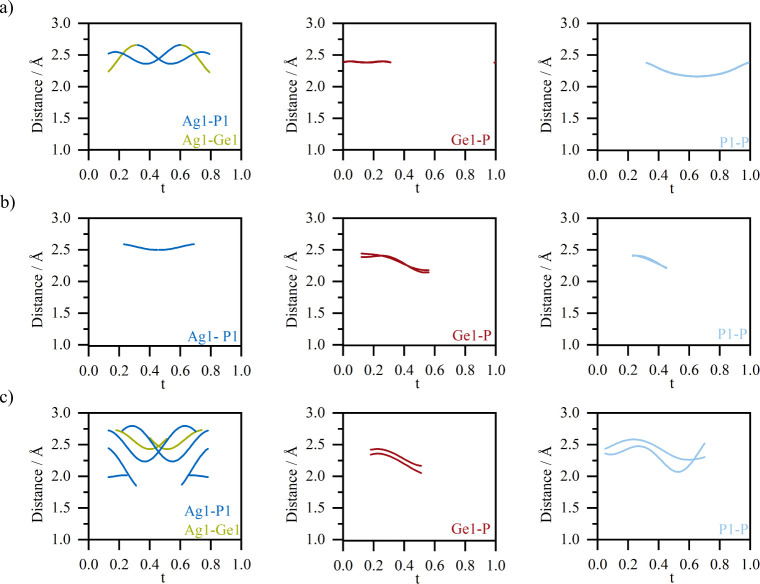
(a) t-plots of the interatomic distances Ag1-**M2**(P1/Ge1) (left), **M2**-P (Ge1–P (middle), P1–P (right)) using the “mirror plane model”. (b) t-plots of the interatomic distances Ag1-**M2**(P1/Ge1) (left), **M2**-P (Ge1–P (middle), P1–P (right)) using the “split atom model”. (c) t-plots of the interatomic distances Ag1-**M2**(P1/Ge1) (left), **M2**-P (Ge1–P (middle), P1–P (right)) using the “crenel model”.

In the “mirror plane model”, the distances to the phosphorus on the **M2** position (Ag–P1) are 2.4–2.6 Å and also Ag–Ge1 distances occur with bond lengths of 2.2–2.7 Å (Figure 6a) left). The “split atom model” shows only Ag–P1 distances that lie in the range between 2.5 and 2.6 Å. However, the “crenel model” shows a larger range of Ag–P1 distances between 1.8–2.8 Å, where the shorter ones are assumed to be too short for the Ag–P bond distance. The most realistic scenario of bond lengths is obtained by the “split-atom model” with the typical Ag–P distances of 2.5–2.8 Å.^17,45,46^

The distances between germanium and the phosphorus backbone in the “mirror plane model” range from 2.37 to 2.41 Å. These values are consistent with reports from literature (Na_10_Ge_2_P_6_ (2.33–2.43 Å),^47^ NaGe_3_P_3_ (2.31–2.45 Å),^48^ GeP (2.34–2.38 Å),^49^ α-/β-Li_8_GeP (2.38–2.44 Å),^50^ Na_2_Ge_3_P_3_ (2.3–2.37 Å).^50^ In both other models, the Ge–P distance range is broader (2.1–2.4 Å). While a distance of 2.45/2.4 Å agrees with the upper limits reported in literature the minimum distance of 2.15/2.1 Å lies below the typical values. So, all three models agree well with literature values in their upper range, while the lower values of the “split atom”- and “crenel model” (<2.31 Å) are slightly below the experimental bond length ranges and, therefore, may be physically less likely.

In the “mirror plane model” a P–P binding range of 2.16–2.40 Å was found which corresponds to the typical bond ranges in the element and in polyphosphides.^10^ The “split atom model” (2.20–2.40 Å) avoids short P–P bonds and remains within the chemically reasonable range of three-bonded phosphorus. The “crenel model”, on the other hand, displays the widest range of 2.10–2.50 Å, including both atypically short and long distances outside the accepted bond lengths range.

A comparison of the de Wolff sections of the three structure models (electron densities as a function of *x*_4_; see Figures S1–S3) shows that the electron density can be described similarly well by all of them.

Considering the interatomic distances within the three structure models, it can be stated that in the “split atom model” and the “mirror plane model” the P–P binding lies in its usual range.^10^ Just the “crenel model” shows rather short and long distances. For the description of the cation substructure being the origin of the incommensurability, the “split atom model” can be regarded as the model with chemically meaningful bond lengths, making it the most plausible model for the structure description.

We were able to identify first- and second-order satellite reflections in the powder diffractogram (Figures S7 and S8). Note that the powder pattern of Ag_1.7(1)_Ge_1.0(1)_P_14_ contains some additional reflections of an unknown side product. Le-Bail fits of the powder diffractogram of Ag_1.7(1)_Ge_1.0(1)_P_14_ leads to the cell parameters *a* = 12.980(1) Å, *b* = 3.2596(3) Å, *c* = 10.844(1) Å, **q** = (0, 0.40, 0) and of Ag_1.4(1)_Sn_1.0(1)_P_14_ to *a* = 13.028(2) Å, *b* = 3.2646(5) Å, *c* = 10.933(1) Å, **q** = (0, 0.41, 0) (Figure S8).

### SEM-EDS Analyses

EDS analyses of the materials show that the ideal **M1**:**M2** ratio in the HgPbP_14_ type materials of 1 is shifted toward a higher silver content (Table 2).

**Table 2:** EDS Results of the Title Compounds Compared to EDS Results of “AgSnP_14_” Reported in Literature and Composition Derived from Single-Crystal X-ray Diffraction Data

Compound		Ag [at%]	Sn/Ge [at%]	P [at%]	Refs
AgGeP_14_	Calculated	6.25	6.25	87.5	
Ag1.7(1)Ge1.0(1)P_14_	From EDS	10(2)	5.6(1)	84(2)	This work
	From XRD	9.9(1)	5.9(1)	84(1)	This work
AgSnP_14_	Calculated	6.25	6.25	87.5	
Ag1.4(1)Sn1.0(1)P_14_	From EDS	10(1)	6(1)	84(1)	This work
	From XRD	8.7(1)	5.9(1)	85(1)	This work
AgSnP_14_		8.8	7.6	83.7	Lange 2006
AgSnP_14_		9.3	11.2	79.5	Eschen 2002

A slight but not significant excess of Ag vs **M2** was already observed in a previous study of “AgSnP_14_”.^30^ In the case of the EDS results reported by Eschen et al., the ratio is inverse to the values found in this study.

Our EDS measurements result in sum formulas of Ag_1.7(1)_Ge_1.0(1)_P_14_ and Ag_1.4(1)_Sn_1.0(1)_P_14_ if the composition is normalized to the phosphorus content of 14 phosphorus atoms per formula unit according to the HgPbP_14_ structure type. Taking into account the rather high systematic errors of EDS measurements on the as-synthesized needles, the obtained values correspond reasonably well to the composition found in the single-crystal structure refinements. Assuming classical oxidation states of + I for Ag and + II for Ge, the composition of Ag_1.7(1)_Ge_1.0(1)_P_14_ from the single crystal refinement does not lead to a charge balanced situation. Taking the nature of the modulated structure into account, it is clear that the real structure is composed by fractions of a [P_15_]^−^ (AgP_15_ fraction), [P_20_]^2–^ (Cu_2_P_20_ fraction) and a [P_14_]^4–^ polyanion substructure that will result in an overall reduced anion charge lower than 4– per formula unit (see Figure 4 for polyanion representations and substitution pattern of different cation and sites). Therefore, according to our determined composition of Ag_1.7(1)_Ge_1.0(1)_P_14_ we would end up with a negative charge of 3.7 for the polyanion substructure. From our structure refinements we can determine the [P14] content which corresponds directly to the Ge content in the refinement (the Ge sof on the **M2** site). The two other fractions where either two Ag^+^ cations occupy the **M1** site (Cu_2_P_20_-like cation arrangement) or one Ag^+^ on **M1** and a P atom on the **M2** site (AgP_15_-like distribution of ions/atoms) are not derivable from our data. Nevertheless, both fractions are integral parts of the present title compounds.

### ^119^Sn Mössbauer Spectroscopy and X-ray Photoelectron Spectroscopy

The ^119^Sn spectrum of Ag_1.4(1)_Sn_1.0(1)_P_14_ measured at 78 K is presented in Figure 7a, along with a transmission integral fit. The corresponding fitting parameters are summarized in Table 3. In accordance with the presence of a single Sn site, Ag_1.4(1)_Sn_1.0(1)_P_14_ shows a single asymmetric quadrupole doublet at an isomer shift of 2.78(1) mm s^–1^ and an experimental line width of 0.91(2) mm s^–1^. The isomer shift value indicates Sn­(II), which is compatible with an ionic formula Ag_1.4_Sn_1.3_P_14_ ≡ 1.4Ag^+^ + 1.3Sn^2+^ + 10P^0^ + 4P^–^ and the crystal structure. The Sn­(II) atoms occupy the mixed-occupied cation position **M2** (occupancy: 0.3 Sn and 0.7 P1), which offers sufficient space for the lone pairs of the Sn­(II) atoms due to the one-sided coordination of the Sn atoms by four phosphorus atoms. The lone-pair character leads to an asymmetric electron density distribution at the Sn nuclei, resulting in an electric quadrupole splitting of 1.59(1) mm s^–1^.

**Figure 7:**
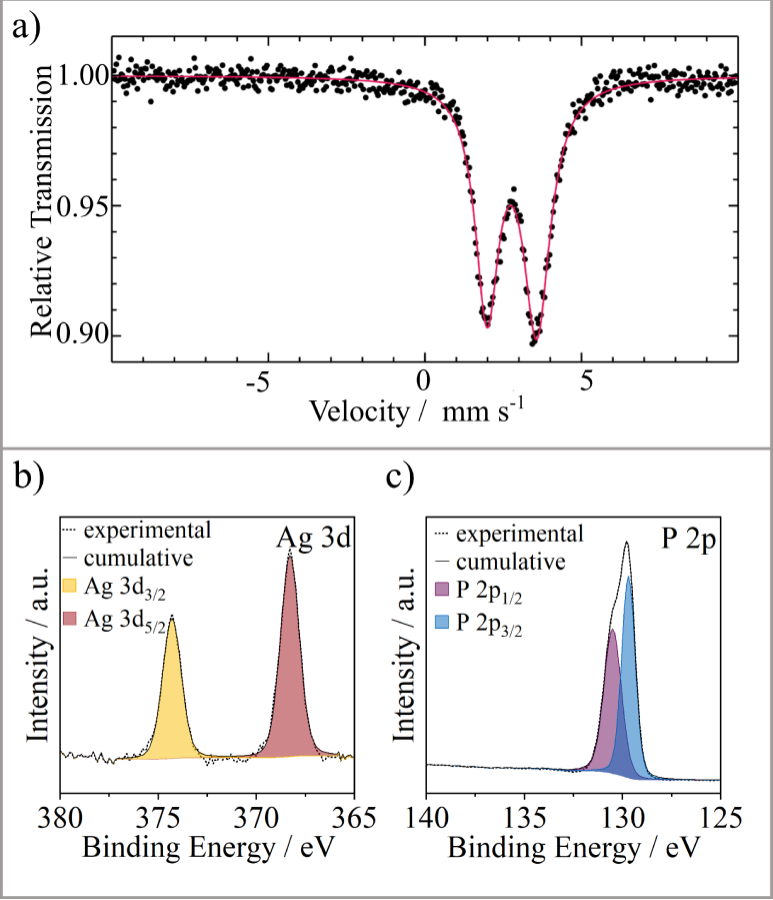
(a) Experimental (data points) and simulated (red line) ^119^Sn Mössbauer spectrum of Ag_1.4(1)_Sn_1.0(1)_P_14_ measured at 78 K. (b) High-resolution Ag 3d XPS spectrum of Ag_1.7(1)_Ge_1.0(1)_P_14_. (c) High-resolution P 2p XPS spectrum of Ag_1.7(1)_Ge_1.0(1)_P_14_.

**Table 3:** Fitting Parameters of the ^119^Sn Mössbauer Spectroscopic Measurement for Ag_1.4(1)_Sn_1.0(1)_P_14_ at 78 K^a^

δ /mm s^–1^	Δ*E*_Q_/mm s^–1^	Γ/mm s^–1^	*A*_21_	*W*_21_
2.78(1)	1.59(1)	0.91(2)	1.24(3)	1.14(3)

a δ = isomer shift, Δ*E*_Q_ = quadrupole splitting, Γ = experimental line width, A21 = area ratio, and W21 = line width ratio of the quadrupole split signal

In 2009, AgSbP_14_ was investigated by ^121^Sb Mössbauer spectroscopy,^37^ where the lone-pair Sb^3+^ cations also occupy the **M2**-position (8*d*; site symmetry 1) and not the tetrahedrally coordinated **M1**-position (4*c*, site symmetry .*m*.). Sn_4_P_3_ is a well-studied tin phosphide because of its application as an anode material for sodium-ion batteries.^51^ The reported isomer shift of 2.67 mm s^–1^ observed for Sn_4_P_3_ is rather similar to the one in our title compound.^52^ The doublet asymmetry in the spectrum can be ascribed to texture effects.^53–56^

X-ray photoelectron spectroscopy (XPS) was performed to get further insight into the oxidation states of Ag_1.7(1)_Ge_1.0(1)_P_14._ Figure 7b shows the high-resolution Ag 3d core level region with the expected 3d_3/2_ 3d_5/2_ doublet peaks. The binding energy of 3d_5/2_ is 367 eV and therefore lies in the usual range for Ag^+^.^57^ The 2p_3/2_ binding energy value for phosphorus is found to be 129.7 eV (Figure 7c). This relates to the polyphosphide groups in the material, so no surface oxidation to P_*x*_O_*y*_ species occurred in the sample, as no P_*x*_O_*y*_ related peaks were observed at >132 eV.^57^ We were not able to distinguish between the different oxidation states of phosphorus in the title compounds. Our sharp signal group corresponds well with covalently bonded phosphorus, for instance found in phosphorus/carbon composite materials for batteries where oxidation states around 0 and – I can be found.^58^

### Raman Spectroscopy

In Figure 8, the two-dimensional polarization-dependent Raman spectra of Ag_1.7(1)_Ge_1.0(1)_P_14_ (a) and Ag_1.4(1)_Sn_1.0(1)_P_14_ (c) in the range of 80–500 cm^–1^ are shown. The angles in the figure indicate that polarization takes place in the direction of the fiber at 0° and perpendicular to the fiber at 90°. In both cases, the most intense Raman signals appear in the fiber direction (approximately 0° and 180°). For Ag_1.7(1)_Ge_1.0(1)_P_14_, the most intense Raman signals in fiber direction are at 94, 140, 185, 230, 316, and 371 cm^–1^. The most intense signal perpendicular to the fiber (90°) lies at 477 cm^–1^. Similar Raman shifts can be observed in Ag_1.4(1)_Sn_1.0(1)_P_14_. They occur at 93, 136, 180, 211, 318, and 366 cm^–1^. As is the case of Ag_1.7(1)_Ge_1.0(1)_P_14_, a signal perpendicular to the fiber direction occurs at 472 cm^–1^. In the related material AgP_15_, the Raman signals could be assigned to the bending and stretching modes of distinct structural units.^17^ Since structural motifs found in the crystal structure of AgP_15_ are also present in the title compounds, a comparison can be made between those materials. Figure 8b,d shows Raman spectra of the title compounds at different angles in comparison to the Raman spectrum of AgP_15_. The dotted lines indicate the most polarization dependent Raman modes. According to the assignment of the Raman bands in AgP_15_, the modes at 94 cm^–1^ (Ag_1.7(1)_Ge_1.0(1)_P_14_) and 93 cm^–1^ (Ag_1.4(1)_Sn_1.0(1)_P_14_) are most likely caused by the vibrations of the Ag–P bonding.

**Figure 8:**
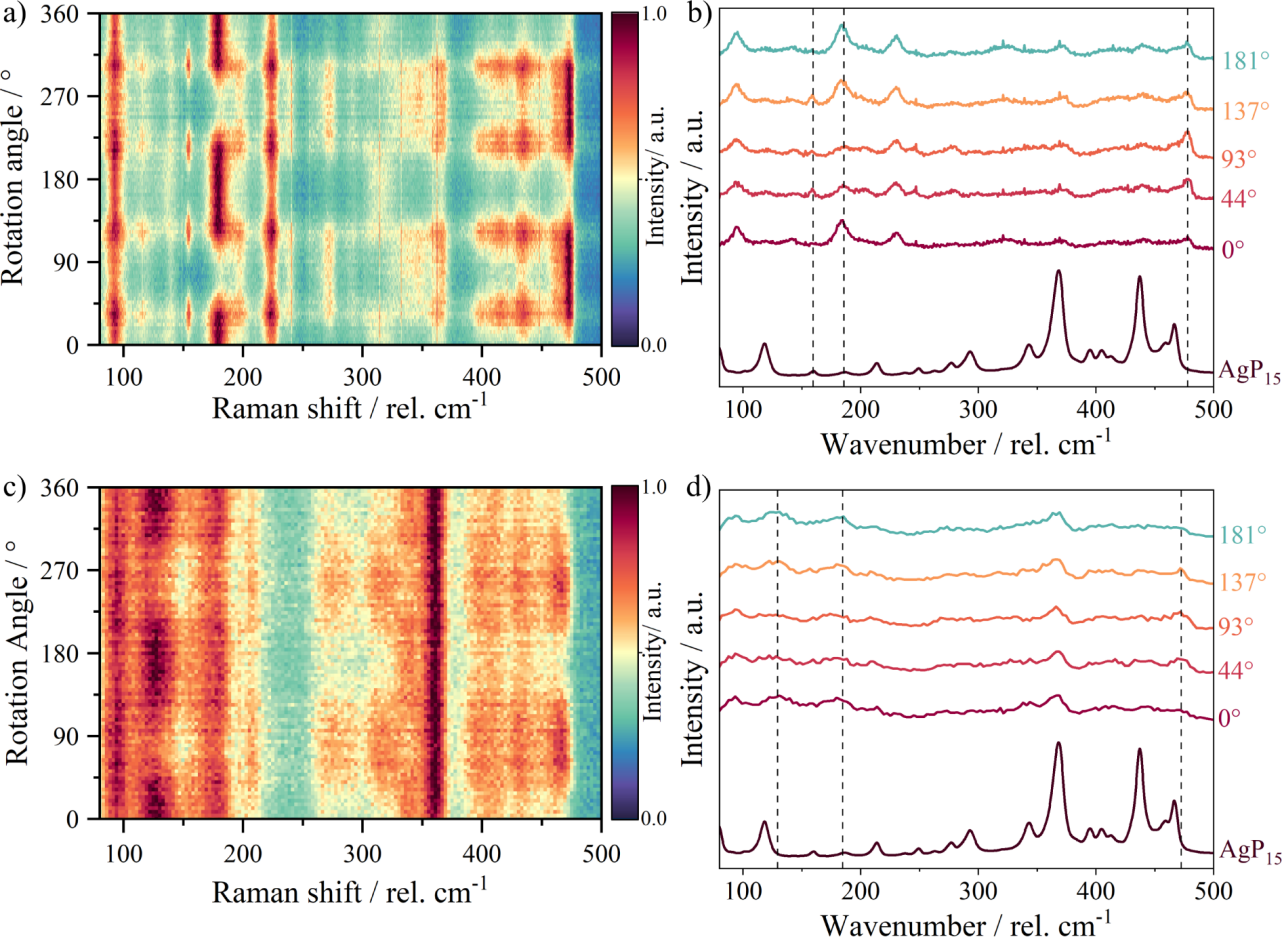
(a) Angle-resolved polarized Raman spectrum between 80 and 500 cm^–1^ of Ag_1.7(1)_Ge_1.0(1)_P_14_. (b) Selected Raman spectra between 80 and 500 cm^–1^ of Ag_1.7(1)_Ge_1.0(1)_P_14_ in comparison to the Raman spectrum of AgP_15_^17^ the dashed lines indicate the most angle-dependent bands. (c) Angle-resolved polarized Raman spectrum between 80 and 500 cm^–1^ of Ag_1.4(1)_Sn_1.0(1)_P_14_. (d) Selected Raman spectra between 80 and 500 cm^–1^ of Ag_1.4(1)_Sn_1.0(1)_P_14_ in comparison to the Raman spectrum of AgP_15_.^17^ The dashed lines indicate the most angle-dependent bands.

The bands between 135 cm^–1^ and 300 cm^–1^ originate from bending vibrations of the *P–P–P* bonds. In AgP_15_, no bands occur between 300 and 350 cm^–1^, whereas a band is clearly visible at 316 cm^–1^ in Ag_1.7(1)_Ge_1.0(1)_P_14_ and 318 cm^–1^ in Ag_1.4(1)_Sn_1.0(1)_P_14_. We assigned this band to Ge–P or Sn–P vibrations, respectively. In SnP, a Sn–P stretching mode was observed at 143 cm^–1^ and was calculated to be at 128 cm^–1^.^59^ The broad mode at about 136 cm^–1^ visible in the Sn but not in the Ge compound is therefore assigned to a Sn–P mode. The bands above 350 cm^–1^ and the inversed intensity of the signals at high Raman shifts are presumably caused by *P–P–P* stretching.^17^

### Photoluminescence and Resistivity Measurements

Figure 9a shows the measured photoluminescence spectrum of Ag_1.7(1)_Ge_1.0(1)_P_14_. The data are fitted using Lorentzian curves, resulting in a good coefficient of determination (*R* ^2^ = 0.9654). The measurement shows four broad photoluminescence signals. The broad signals have their maximum at 2.00 eV (peak 1), 1.90 eV (peak 2), 1.80 eV (peak 3), and 1.70 eV (peak 4). Compared to that, the measured photoluminescence spectrum of Ag_1.4(1)_Sn_1.0(1)_P_14_ (Figure 9b) shows only three broad photoluminescence signals. Their position is identical to that of the broad signals in Ag_1.7(1)_Ge_1.0(1)_P_14_ as they are visible at 2.02 eV­(peak 1), 1.90 eV (peak 2), and 1.80 eV (peak 3). The values are blue-shifted compared to the band gap of the related compound ZnSnP_14_ (1.6 ± 0.1 eV). This tendency is consistent among other main group semiconductors when substituting with a more electronegative element. In this case, silver possesses a higher electronegativity than zinc; therefore, the energy difference between the valence and conduction band edges is increased.

**Figure 9:**
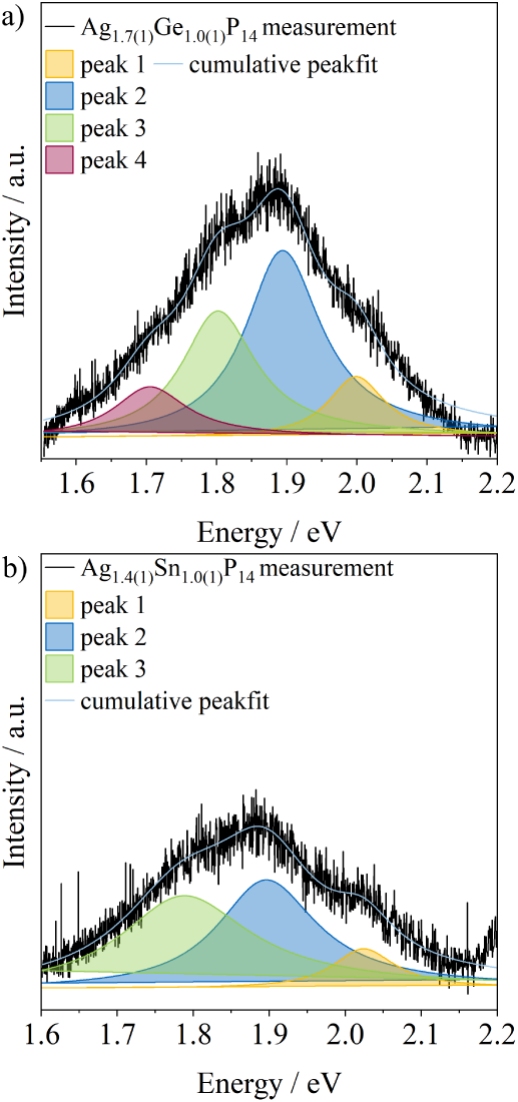
(a) Photoluminescence spectrum of Ag_1.7(1)_Ge_1.0(1)_P_14_. The measured intensity (black) was fitted using four Lorentzian curves (red, green, blue, yellow), and the resulting cumulative peak fit is indicated by a light blue line. (b) Photoluminescence spectrum of Ag_1.4(1)_Sn_1.0(1)_P_14_. The measured intensity (black) was fitted using three Lorentzian curves (green, blue, yellow), and the resulting cumulative peak fit is indicated by a light blue line. The data is fitted using Lorentzian curves, resulting in a decent coefficient of determination (*R* ^2^ = 0.928).

In addition, we could determine the resistivity of a Ag_1.7(1)_Ge_1.0(1)_P_14_ single crystal (5.11 Ωcm) at ambient temperature. This corresponds to a conductivity of 0.2 S/cm, which is in the range of classical semiconductors like boron phosphide (*p*-type: 0.4–2 S/cm; *n*-type: 0.3–0.6 S/cm) or gallium phosphide (0.15–0.9 S/cm).^60^ This value can also be compared to the resistivities of microcrystalline HgPbP_14_ (6 × 10^4^ Ωcm) and ZnSnP_14_ (3 × 10^5^ Ωcm)^28^. The difference (single-crystal vs microcrystalline bulk) in the acquisition of the data may, however, bias the obtained resistivity values. A determination of the resistivity of Ag_1.6_Sn_1.2_P_14_ was not successful due to surface oxidation of the compound and the necessary treatment in air prior to the measurement.

In HgPbP_14_-type compounds (**(M1)­(M2)**P_14_) that are formed by two M^2+^ cations, like HgPbP_14_ itself, HgSnP_14_, ZnPbP_14_ and CdPbP_14_,^26,29^ the crystal structure is ordered with no occupancy or displacement disorder (see Figure 5b,d). We classify them as M^2+^(on **M1** site)-M^2+^(on **M2** site) compounds. **M1** and **M2** sites are fully occupied by the cations and one observes no need to create split positions. If cations with a charge different from +II are incorporated, other arrangements are found: Lone pair cations like Sn^2+^ or Sb^3+^ tend to occupy the **M2** site within the polyphosphide moiety that offers enough space to accommodate the stereoactive lone-pair cation. In the title compounds this gives rise to the observed [P2**M2**] subunits. One example for an ordered arrangement of Sb^3+^ on the **M2** site and Ag^+^ on the **M1**, respectively, is AgSbP_14_.^30^ Also in this case of an M^+^(**M1** site)-M^3+^(**M2** site) type compounds, the most likely structure is that of fully ordered HgPbP_14_.

The situation changes if one now offers cations that can in principle adopt more than one oxidation state in the present chemical environment, for instance a M^1+^/M^3+^ cation (e.g., Au) or M^2+^/M^4+^ (Ge, Sn, Pb). That would result in M^+^/M^3+^(**M1** site)-M^2+^M^4+^(**M2** site) compounds or even more complex systems where all cations may be located on both sites. Such examples are known, e.g., Cu_0.73_Sn_1.27_P_14_ ^30^ and Au_0.64_Sn_1.36_P_14_.^29^ In Cu_0.73_Sn_1.27_P_14_, the **M1** position is occupied by Cu^+^ while for Au_0.64_Sn_1.36_P_14_ the question arose if Au^+^ and Au^3+^ is present on that site. In both compounds, the **M1** and the **M2** positions are occupied by Sn^2+^ to a certain amount, which results in a significantly larger Sn content in the compounds (Sn content >1 per formula unit). The lone pair active Sn^2+^ ion is not only occupying the **M2** site within the [P2**M2**] moiety but also the tetrahedrally surrounded **M1** site that connects the different strands within the crystal structure (see Figure 5). The occurrence of Sn^4+^ in those two compounds was rejected due to ^119^Sn Mössbauer spectroscopic investigations, whereas the question of Au^3+^ incorporation is still open.^30,37^ Despite this complex question concerning the oxidation states of the cations, both compounds adopt the same structure, a HgPbP_14_-type one where the **M1** position is occupied by Cu^+^, respectively (Au^+^, Au^3+^) and the **M2** position is split and accommodates the lone pair Sn^2+^ cation in both cases. Cu_0.73_Sn_1.27_P_14_ resembles a M^+^/M^2+^(**M1** site)-M^2+^(**M2** site) compound where Sn^2+^ has to occupy both sites to allow overall charge balancing. Au_0.64_Sn_1.36_P_14_ is most likely of the same M^+^/M^2+^(**M1** site)-M^2+^(**M2** site) type because a mixed occupancy of Au^+^, Sn^2+^ and Au^3+^ on the same **M1** site is rather unlikely.

Even in these two cases, a commensurate structure model (excluding cell enlargement and super structure) is sufficient to describe the crystal structure properly. If we now introduce silver as M^+^ and a M^2+^/M^4+^ couple to the system, the situation changes. Ag^+^ also occupies the **M1** site and Sn is only incorporated in form of a Sn^2+^ ion, as substantiated by ^119^Sn Mössbauer spectroscopy. Taking the compositions of the title compounds of Ag_1.4(1)_Sn_1.0(1)_P_14_ and Ag_1.7(1)_Ge_1.0(1)_P_14_ into account and comparing them with the previously mentioned Cu_0.73_Sn_1.27_P_14_ and Au_0.64_Sn_1.36_P_14_, we observe a drastic change in the compositional parameters within the cation substructure. The late transition metal content significantly exceeds 1 while the Sn/Ge content is now only 1 per formula unit. To achieve a charge balanced compound, one phosphorus site of the [P3] unit (and also the **M2** site of the [P2**M2**] unit, as illustrated in Figure 5, average structure) have to be described by an incommensurate position and occupancy modulation to allow a physically meaningful (and in consequence overall charge balanced) structure model. The title compounds are therefore the missing link between the fully ordered M^2+^(**M1** site)-M^2+^(**M2** site) and the (M^+^,M^2+^)-(**M1** site)- M^2+^(**M2** site) systems. Now these two title compounds are (M^+^)-(**M1** site)-(M^2+^/P^–^)­(**M2** site) systems where the **M2** site is incommensurably occupied by either P^–^ (resulting in a [P3] entity) or M^2+^ (now creating a [P2**M2]** unit). In brief, the structure is determined by a superposition of fragments of Cu_2_P_20_, AgP_15_, and HgPbP_14_-like entities.

The crystal quality (for the determination of the crystal structure) reduced drastically from the fully ordered HgPbP_14_ M^2+^(**M1** site)-M^2+^(**M2** site) compounds, via the M^+^/M^2+^(**M1** site)-M^2+^(**M2** site) ones that show split positions for the **M2** site, to the title compounds that display incommensurate disorder phenomena. On the other hand, as illustrated by SEM experiments the delamination tendency seems to increase drastically in the same order so that the tile compounds are intriguing compounds for the preparation of quasi-1D nanomaterials. Experiments are currently underway and will be reported in an upcoming study.

## Conclusions

The compounds Ag_1.4(1)_Sn_1.0(1)_P_14_ and Ag_1.7(1)_Ge_1.0(1)_P_14_ with the refined unit cell compositions Ag_2.2(1)_Ge_1.3(1)_P_18.7(1)_ and Ag_1.9(1)_Sn_1.3(1)_P_18.7(1)_ (*Z* = 1) are two representatives of the Ag–Sn–P and Ag–Ge–P systems adopting crystal structures closely related to the HgPbP_14_ type. The germanium compound is the second known ternary material in the Ag–Ge–P phase diagram. Both compounds show a complex incommensurately modulated structure with an occupancy and displacive modulation within the cation substructure. ^119^Sn Mössbauer spectroscopy shows that tin occurs in the oxidation state +II, supporting the postulated structure model. XPS illustrates that the surface oxygen contamination is low and no significant amount of phosphate is present. Angle-resolved Raman spectroscopy illustrates the anisotropy of the material and the close relation between of the modulated anion substructure to semiconducting AgP_15_. It similarly emphasizes the anisotropic characteristics of the materials. Photoluminescence shows band gaps around 1.9 eV, which is blue-shifted in relation to other representatives of the structural family and characterizes the materials as semiconductors. Furthermore, we were able to substantiate the semiconducting behavior of Ag_1.7(1)_Ge_1.0(1)_P_14_ via single-crystal resistivity measurements. This, along with their needle-shaped morphology, makes them interesting for potential applications in optical and electronic devices.

## Supplementary Material



## Notes

Additional crystallographic information, such as De-Wolff sections; additional structure refinement data, including tables of atomic coordinates, displacement parameters, and figures of powder XRD data; and resistivity data are denoted in the Supplement (PDF)

## Abbreviations

1D: one-dimensional
2D: two-dimensional
EDS: energy-dispersive X-ray spectroscopy
SE-SEM: secondary electron scanning electron microscopy
XRD: X-ray diffraction
XPS: X-ray photoelectron spectroscopy
