# Supplementary material for: Low-Dimensional Semiconducting Silver (Germanium, Tin) Polyphosphides – Incommensurately Modulated Derivates of the HgPbP14 Structure Type
Source: Inorg Chem. 2025 Sep 10;64(37):19055–67. doi: 10.1021/acs.inorgchem.5c03307 (PMC12458684; doi:10.1021/acs.inorgchem.5c03307)
Supplement: Supplementary file 1 [file ic5c03307_si_001.pdf]

# *Supporting Information* for Low-dimensional Semiconducting Silver (Germanium, Tin) Polyphosphides – incommensurately-modulated derivates of the $HgPbP_{14}$ structure type

*Kathrin Vosseler [1], Aylin Koldemir[2], Rainer Pöttgen[2], Thomas Doert[3], Tom Nilges[*,1]*

AUTHOR ADDRESS

1 Synthesis and Characterization of Innovative Materials, TUM School of Natural Sciences, Department of Chemistry, Technical University of Munich, Lichtenbergstraße 4, 85748 Garching b. München, Germany

2 Institut für Anorganische und Analytische Chemie, Universität Münster, Corrensstraße 30, 48149 Münster, Germany

3 Fakultät Chemie und Lebensmittelchemie, Technische Universität Dresden, 01062 Dresden, Germany

*corresponding author: tom.nilges@tum.de

## 1. Literature list for binary and ternary Ag-Ge-P/ Ag-Sn-P phases:

**GeP:**

Donohue, P. C.; Young, H. S. Synthesis, structure, and superconductivity of new high-pressure phases in the systems Ge-P and Ge-As. *J. Solid State Chem.* **1970**, *1* (2), 143-149. DOI: https://doi.org/10.1016/0022-4596(70)90005-8.

**$GeP_2$:**

Osugi, J.; Namikawa, R.; Tanaka, Y. Chemical reaction at high temperature and high pressure IV: high pressure phases in the Ge-P system. *Rev. Phys. Chem. Jpn.* **1967**, *37* (2), 81-93.

**$GeP_3$:**

Gullman, J.; Olofsson, O. The crystal structure of $SnP_3$ and a note on the crystal structure of $GeP_3$. *J. Solid State Chem.* **1972**, *5* (3), 441-445. DOI: https://doi.org/10.1016/0022-4596(72)90091-6.

Donohue, P. C.; Young, H. S. Synthesis, structure, and superconductivity of new high pressure phases in the systems Ge-P and Ge-As. *J. Solid State Chem.* **1970**, *1* (2), 143-149. DOI: https://doi.org/10.1016/0022-4596(70)90005-8.

**$SnP_3$/ $Sn_4P_3$:**

Olofsson, O.; Aava, U.; Haaland, A.; Resser, D.; Rasmussen, S.; Sunde, E.; Sørensen, N. A. X-ray investigation of the tin-phosphorus system. *Acta Chem. Scand.* **1970**, *24* (4), 1153-1162.

Gullman, J.; Olofsson, O. The crystal structure of $SnP_3$ and a note on the crystal structure of $GeP_3$. *J. Solid State Chem.* **1972**, *5* (3), 441-445. DOI: https://doi.org/10.1016/0022-4596(72)90091-6.

**SnP:**

Donohue, P. C. Synthesis, structure, and superconducting properties of new high-pressure forms of tin phosphide. *Inorg. Chem.* **1970**, *9* (2), 335-337.

Gullman, J. The crystal structure of SnP. *J. Solid State Chem.* **1990**, *87* (1), 202-207. DOI: https://doi.org/10.1016/0022-4596(90)90083-A.

**$Sn_3P_4$:**

Zaikina, J. V.; Kovnir, K. A.; Sobolev, A. N.; Presniakov, I. A.; Kytin, V. G.; Kulbachinskii, V. A.; Olenev, A. V.; Lebedev, O. I.; Tendeloo, G. V.; Dikarev, E. V.; et al. Highly Disordered Crystal Structure and Thermoelectric Properties of $Sn_3P_4$. *Chem. Mater.* **2008**, *20* (7), 2476-2483.

**$Ag_3Sn$:**

Fairhurst, C.; Cohen, J. The crystal structures of two compounds found in dental amalgam: $Ag_2Hg_3$ and $Ag_3Sn$. *Struct. Sci.* **1972**, *28* (2), 371-378.

**$Ag_{0.8}Sn_{0.2}$:**

Shalaby, R. M. Effect of rapid solidification on mechanical properties of a lead free Sn–3.5Ag solder. *J. Alloys Compd.* **2010**, *505* (1), 113-117. DOI: https://doi.org/10.1016/j.jallcom.2010.05.179.

**$Ag_{0.9}Sn_{0.1}$:**

Melnychenko-Koblyuk, N.; Romaka, V.; Romaka, L.; Stadnyk, Y. Interaction between the components in the {Zr, Hf}-Ag-Sn ternary systems. *Chem. Met. Alloys* **2011**, *(4)*, 234-242.

**$AgP_{15}$:**

Grotz, C.; Schäfer, K.; Baumgartner, M.; Weihrich, R.; Nilges, T. One-Dimensional $[P15]^-$ Tubes in Layered Semiconducting $AgP_{15}$. *Inorg. Chem.* **2015**, *54* (22), 10794-10800.

**$Ag_3P_{11}$:**

Möller, M. H.; Jeitschko, W. Preparation and crystal structure of trisilver undecaphosphide, $Ag_3P_{11}$, an unusual defect tetrahedral compound. *Inorg. Chem.* **1981**, *20* (3), 828-833. DOI: 10.1021/ic50217a039.

**$AgP_2$:**

Olofsson, O. The Crystal Structures of $CuP_2$ and $AgP_2$ with some Phase Analytical Data of the Cu-P and Ag-P Systems. *Acta Chem. Scand* **1965**, *19* (1), 229-241.

**$Ag_6Ge_{10}P_{12}$:**

Von Schnering, H.; Häusler, K. $Ag_6Ge_{10}P_{12}$, eine Verbindung mit $Ag_6$-Cluster. *Revue de Chimie minérale* **1976**, *13*, 71-81.

**$Ag_3SnP_7$:**

Shatruk, M. M.; Kovnir, K. A.; Shevelkov, A. V.; Popovkin, B. A. $Ag_3SnP_7$: A polyphosphide with a unique $(P_7)$ chain and a novel $Ag_3Sn$ heterocluster. *Angew. Chem. Int. Ed.* **2000**, *39* (14), 2508-2509.

# 2. Further Single crystal XRD data of $Ag_{1.7(1)}Ge_{1.0(1)}P_{14}$ and $Ag_{1.4(1)}Sn_{1.0(1)}P_{14}$ at room temperature

**Table S1.** Further Single crystal XRD data of $Ag_{1.7(1)}Ge_{1.0(1)}P_{14}$ and $Ag_{1.4(1)}Sn_{1.0(1)}P_{14}$ at room temperature.

| | $Ag_{1.7(1)}Ge_{1.0(1)}P_{14}$ | $Ag_{1.7(1)}Ge_{1.0(1)}P_{14}$ | $Ag_{1.4(1)}Sn_{1.0(1)}P_{14}$ |
|---|---|---|---|
| refined composition | $Ag_{2.2(1)}Ge_{1.4(1)}P_{18.6(1)}$ | $Ag_{2.2(1)}Ge_{1.2(1)}P_{18.8(1)}$ | $Ag_{1.9(1)}Sn_{1.3(1)}P_{18.7(1)}$ |
| molar mass (g mol$^{-1}$) | 917.7 | 909.7 | 935.1 |
| Modulation model | Split, crenel | Mirror plane, sines | Mirror plane, sines |
| modulation model | needle / black | | |
| crystal shape/color | Orthorhombic | | |
| crystal system | *Pnma*(0*β*0)s00 | | |
| Z (per unit cell) | 1 | | |
| *a* (Å) | 12.9856(14) | | 13.0139(14) |
| *b* (Å) | 3.2648(4) | | 3.2602(4) |
| *c* (Å) | 10.8410(12) | | 10.9053(12) |
| *V* (Å$^3$) | 459.61(9) | | 462.69(9) |
| **q** vector | 0.39 | | 0.42 |
| $\rho_{calc.}$ (g cm$^{-3}$) | 3.32 | | 3.35 |
| Diffractometer | Bruker Apex II | | STOE StadiVari |
| Radiation (Å) | 0.71073 (Mo $K_{\alpha1/2}$) | | |
| μ (cm$^{-1}$) | 6.2 | 5.9 | 5.3 |
| F(000) | 428 | 425 | 430 |
| θ range (°) | 2.33 – 32.6 | | 2.12 - 44.82 |
| hkl range | –19/+19, –5/+5, –16/+16 | | –19/+19, –5/+5, –16/+15 |
| no. of reflections | 31918 | | 37585 |
| $R_{int}$ | 0.166 | | 0.128 |
| data/parameters | 1678 / 80 | 1678 / 75 | 1686 / 92 |
| R / wR [I > 3σ (I)] (all) | 0.0412 / 0.0883 | 0.0784 / 0.1817 | 0.0741 / 0.1480 |
| R / wR [all] (all) | 0.0689 / 0.0933 | 0.1110 / 0.1871 | 0.1379 / 0.1639 |
| R / wR [I > 3σ (I)] (main) | 0.0322 / 0.0792 | 0.0627 / 0.1675 | 0.0353 / 0.0697 |
| R / wR [all] (main) | 0.0419 / 0.0808 | 0.0749 / 0.1704 | 0.0575 0.0753 |
| R / wR [I > 3σ (I)] (satellites) | 0.0567 / 0.1011 | 0.1063 / 0.2027 | 0.1484 / 0.2674 |
| R / wR [all] (satellites) | 0.1114 / 0.1103 | 0.1687 /0.2116 | 0.2513 / 0.2951 |
| goodness of fit | 2.14 | 4.28 | 1.56 |
| res. elec. dens. max / min (e Å$^{-3}$) | – 0.94 / + 1.29 | – 2.40 / + 4.61 | –1.98/ +2.11 |

## **3.** Crystal structure parameters and atomic displacements of $Ag_{1.7(1)}Ge_{1.0(1)}P_{14}$

### 3.1. Using the **split atom** model

**Table S2.** Occupancies and modulation functions of the refinement of $Ag_{1.7(1)}Ge_{1.0(1)}P_{14}$ (**split atom** model).

| atoms | site occupancy | ADP type | occupational modulation | positional modulation | ADP modulation |
|---|---|---|---|---|---|
| Ag1 | 0.2796 | aniso | 2 harmonics | 1 harmonics | - |
| Ge1_P1 / P1_Ge1 | 0.1683(14)/ 0.3316 | aniso | 1 harmonics | 2 harmonics | - |
| P2 | 0.5 | aniso | - | 1 harmonics | 1 harmonics |
| P3 | 0.5 | aniso | - | 1 harmonics | 1 harmonics |
| P4 | 0.5 | aniso | - | 1 harmonics | 1 harmonics |
| P5 | 0.5 | aniso | - | 1 harmonics | 1 harmonics |

**Table S3.** Coefficients of the occupational modulation functions of the refinement of $Ag_{1.7(1)}Ge_{1.0(1)}P_{14}$ (**split atom** model).

| function | Ag1 | Ge1_P1/P1_Ge1 |
|---|---|---|
| sin1 | -0.4756(13) | 0.149 |
| cos1 | 0 | -0.1743 |
| sin2 | 0 | - |
| cos2 | 0.340(12) | - |

**Table S4.** Wyckoff positions, atomic coordinates, s.o.f. and anisotropic displacement parameters of $Ag_{1.7(1)}Ge_{1.0(1)}P_{14}$ (**split atom** model).

| Atom | Wyck. | *x* | *y* | *z* | s.o.f. | $U_{eq}/U_{iso}$ | $U_{11}$ | $U_{22}$ | $U_{33}$ | $U_{12}$ | $U_{13}$ | $U_{23}$ |
|---|---|---|---|---|---|---|---|---|---|---|---|---|
| Ag1 | 4*c* | 0.17958(11) | $\frac{3}{4}$ | 0.4824(2) | 0.2796 | 0.02408(16) | 0.0204(2) | 0.0321(3) | 0.0197(3) | 0 | 0.0034(2) | 0 |
| Ge1_P1/ P1_Ge1 | 8*d* | 0.14761(6) | 0.1218(2) | 0.62702(6) | 0.1683(14)/ 0.3316 | 0.0153(2) | 0.0214(4) | 0.0120(3) | 0.0127(3) | 0.0015(3) | -0.0019(3) | -0.0017(3) |
| P2 | 4*c* | 0.32574(7) | $\frac{3}{4}$ | 0.34249(8) | 0.5 | 0.0151(2) | 0.0186(5) | 0.0098(4) | 0.0169(4) | 0 | 0.0046(3) | 0 |
| P3 | 4*c* | 0.02962(7) | $\frac{3}{4}$ | 0.33863(8) | 0.5 | 0.0153(2) | 0.0191(4) | 0.0106(4) | 0.0162(5) | 0 | -0.0054(3) | 0 |
| P4 | 4*c* | 0.06349(7) | $\frac{1}{4}$ | 0.20791(9) | 0.5 | 0.0154(2) | 0.0184(4) | 0.0078(4) | 0.0200(4) | 0 | -0.0028(4) | 0 |
| P5 | 4*c* | 0.42263(7) | $\frac{1}{4}$ | 0.41480(8) | 0.5 | 0.0149(2) | 0.0229(5) | 0.0075(4) | 0.0142(4) | 0 | 0.0039(4) | 0 |

**Table S5.** Coefficients of the positional modulation functions of the refinement of $Ag_{1.7(1)}Ge_{1.0(1)}P_{14}$ (**split atom** model).

| function | axis | Ag1 | Ge1_P1/P1_Ge1 | P2 | P3 | P4 | P5 |
|---|---|---|---|---|---|---|---|
| sin1 | *x* | 0.0034(2) | 0.0029 | -0.00386(10) | -0.00149(10) | -0.00624(10) | -0.00495(11) |
| | *y* | 0 | 0 | 0 | 0 | 0 | 0 |
| | *z* | 0.4824(2) | 0.0051 | -0.00610(12) | 0.00560(12) | 0.00808(12) | -0.00931(12) |
| cos1 | *x* | 0 | 0.0069 | 0 | 0 | 0 | 0 |
| | *y* | 0.0292(3) | -0.0385 | -0.0226(4) | -0.0247(4) | 0.0038(4) | 0.0060(4) |
| | *z* | 0 | -0.0151 | 0 | 0 | 0 | 0 |
| sin2 | *x* | - | -0.0017 | - | - | - | - |
| | *y* | - | 0.0345 | - | - | - | - |
| | *z* | - | 0.01 | - | - | - | - |
| cos2 | *x* | - | 0 | - | - | - | - |
| | *y* | - | 0.0113 | - | - | - | - |
| | *z* | - | 0 | - | - | - | - |

**Table S6.** Coefficients of the ADP modulation functions of the refinement of $Ag_{1.7(1)}Ge_{1.0(1)}P_{14}$ (**split atom** model).

| function | ADP parameter | P2 | P3 | P4 | P5 |
|---|---|---|---|---|---|
| sin1 | $U_{11}$ | 0.0040(7) | 0.0043(7) | -0.0020(7) | -0.0006(8) |
| | $U_{22}$ | -0.0034(6) | 0 | 0 | 0 |
| | $U_{33}$ | 0.0042(7) | 0.0045(7) | -0.0038(7) | -0.0023(6) |
| | $U_{12}$ | 0 | 0 | 0 | 0 |
| | $U_{13}$ | 0.0032(6) | -0.0043(6) | 0.0040(6) | -0.0024(6) |
| | $U_{23}$ | 0 | 0 | 0 | 0 |
| cos1 | $U_{11}$ | 0 | 0 | 0 | 0 |
| | $U_{22}$ | 0 | 0 | 0 | 0 |
| | $U_{33}$ | 0 | 0 | 0 | 0 |
| | $U_{12}$ | -0.0024(6) | -0.0024(6) | 0 | 0 |
| | $U_{13}$ | 0 | 0 | 0 | 0 |
| | $U_{23}$ | 0.0016(6) | 0.0016(6) | 0.0008(6) | 0 |

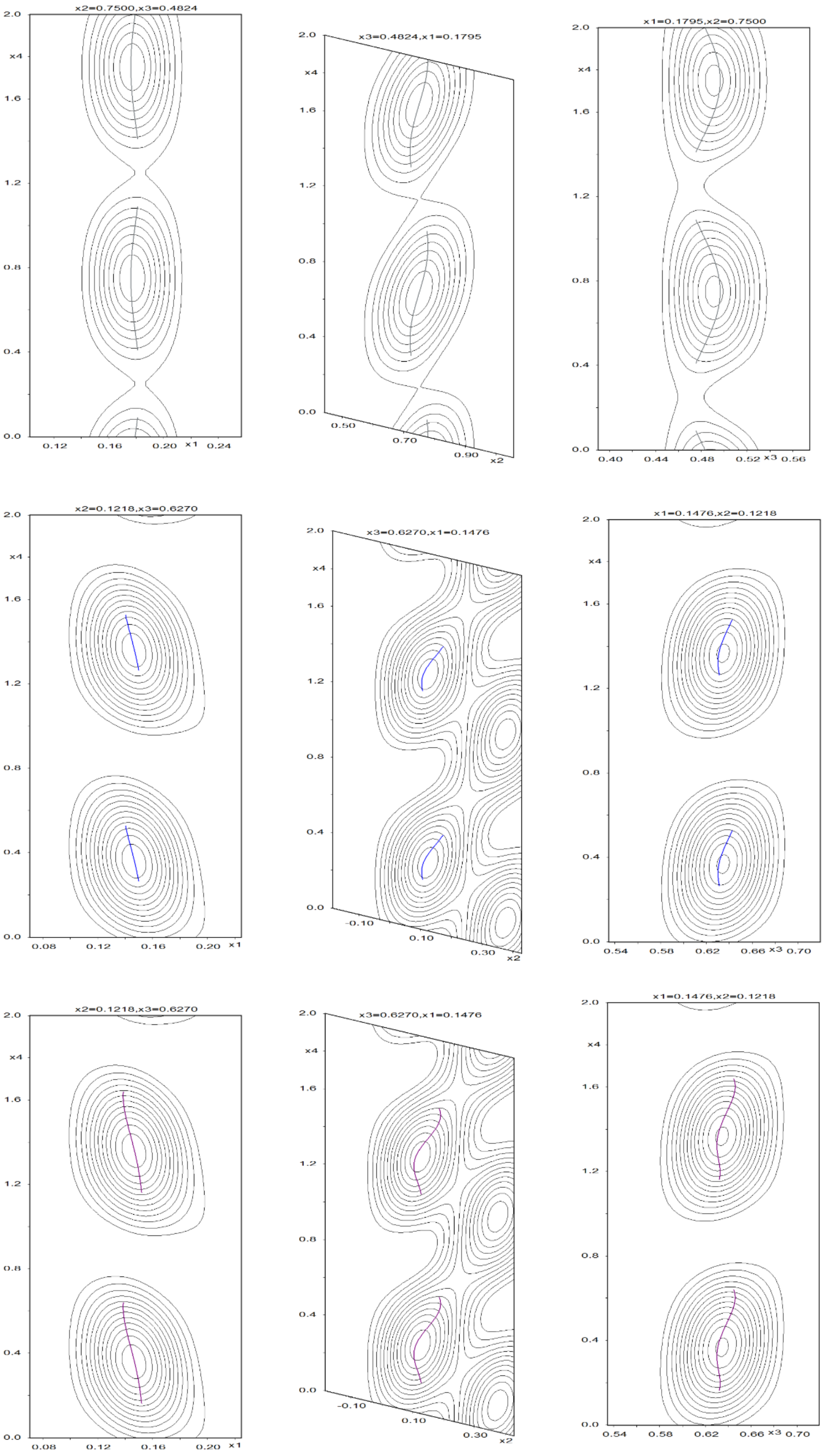


**Figure S1.** De-Wolff sections of $Ag_{1.7(1)}Ge_{1.0(1)}P_{14}$ (**split atom** model). First row: Fourier map (*x*1-*x*4, *x*2-*x*4 and *x*3-*x*4) sections around the Ag1 position; Second row: Fourier map (*x*1-*x*4, *x*2-*x*4 and *x*3-*x*4) sections around the P1 position, Fourier map (*x*1-*x*4, *x*2-*x*4 and *x*3-*x*4) sections around the Ge1 position.

## 3.2. Using the **crenel** model

**Table S7.** Occupancies and modulation functions of the refinement of $Ag_{1.7(1)}Ge_{1.0(1)}P_{14}$ (**crenel** model)

| atoms | site occupancy | ADP type | Δ | $x_4^0$ | occupational modulation | positional modulation | ADP modulation |
|---|---|---|---|---|---|---|---|
| Ag1 | 0.2796 | aniso | - | - | 2 harmonics | 1 harmonics | - |
| Ge1 | 0.1721(14) | iso | 0.344(3) | 0.4 | 1 harmonics | 2 harmonics | - |
| P1 | 0.3279 | iso | 0.656(3) | 0.4364 | 1 harmonics | 2 harmonics | - |
| P2 | 0.5 | aniso | - | - | - | 1 harmonics | 1 harmonics |
| P3 | 0.5 | aniso | - | - | - | 1 harmonics | 1 harmonics |
| P4 | 0.5 | aniso | - | - | - | 1 harmonics | 1 harmonics |
| P5 | 0.5 | aniso | - | - | - | 1 harmonics | 1 harmonics |

**Table S8.** Coefficients of the occupational modulation functions of the refinement of $Ag_{1.7(1)}Ge_{1.0(1)}P_{14}$ (**crenel** model)

| function | Ag1 |
|---|---|
| sin1 | -0.476(2) |
| cos1 | 0 |
| sin2 | 0 |
| cos2 | 0.347(16) |

**Table S9.** Wyckoff positions, atomic coordinates, s.o.f. and anisotropic displacement parameters of $Ag_{1.7(1)}Ge_{1.0(1)}P_{14}$ (**crenel** model)

| Atom | Wyck. | *x* | *y* | *z* | s.o.f. | $U_{eq}/U_{iso}$ | $U_{11}$ | $U_{22}$ | $U_{33}$ | $U_{12} = U_{23}$ | $U_{13}$ |
|---|---|---|---|---|---|---|---|---|---|---|---|
| Ag1 | 4*c* | 0.17958(14) | $\frac{3}{4}$ | 0.4824(3) | 0.2791(10) | 0.0235(2) | 0.0200(3) | 0.0316(4) | 0.0190(5) | 0 | 0.0034(3) |
| Ge1 | 8*d* | 0.1333(2) | 0.1443(7) | 0.6339(3) | 0.1721(14) | 0.011521 | - | - | - | - | - |
| P1 | 8*d* | 0.1505(7) | 0.1518(18) | 0.6237(7) | 0.3279 | 0.0046(10) | - | - | - | - | - |
| P2 | 4*c* | 0.32574(9) | $\frac{3}{4}$ | 0.34250(11) | 0.5 | 0.0147(3) | 0.0183(6) | 0.0094(6) | 0.0165(6) | 0 | 0.0048(5) |
| P3 | 4*c* | 0.02963(10) | $\frac{3}{4}$ | 0.33864(11) | 0.5 | 0.0151(3) | 0.0185(6) | 0.0110(5) | 0.0159(6) | 0 | -0.0054(5) |
| P4 | 4*c* | 0.06344(9) | $\frac{1}{4}$ | 0.20793(12) | 0.5 | 0.0152(3) | 0.0174(6) | 0.0085(5) | 0.0196(6) | 0 | -0.0029(5) |
| P5 | 4*c* | 0.42263(9) | $\frac{1}{4}$ | 0.41475(11) | 0.5 | 0.0144(3) | 0.0221(6) | 0.0067(5) | 0.0144(6) | 0 | 0.0038(5) |

**Table S10**. Coefficients of the positional modulation functions of the refinement of $Ag_{1.7(1)}Ge_{1.0(1)}P_{14}$ (**crenel** model)

| function | axis | Ag1 | Ge1 | P1 | P2 | P3 | P4 | P5 |
|---|---|---|---|---|---|---|---|---|
| sin1 | *x* | 0.0033(3) | 0.0093 | 0.0100(8) | -0.00383(14) | -0.00139(14) | -0.00618(13) | -0.00510(14) |
| | *y* | 0 | 0 | 0 | 0 | 0 | 0 | 0 |
| | *z* | -0.0144(6) | -0.0009 | 0.0060(9) | -0.00587(16) | 0.00559(16) | 0.00809(17) | -0.00922(16) |
| cos1 | *x* | 0 | -0.001 | -0.0035(10) | 0 | 0 | 0 | 0 |
| | *y* | 0.0296(4) | 0.019 | -0.003(3) | -0.0217(5) | -0.0238(5) | 0.0031(5) | 0.0054(5) |
| | *z* | 0 | -0.0088 | -0.0167(11) | 0 | 0 | 0 | 0 |
| sin2 | *x* | - | -0.0025 | -0.0019(11) | - | - | - | - |
| | *y* | - | 0.0037 | -0.022(3) | - | - | - | - |
| | *z* | - | 0.0081 | 0.0194(10) | - | - | - | - |
| cos2 | *x* | - | 0 | 0 | - | - | - | - |
| | *y* | - | 0.0707 | 0.064(3) | - | - | - | - |
| | *z* | - | 0 | 0 | - | - | - | - |

**Table S11.** Coefficients of the ADP modulation functions of the refinement of $Ag_{1.7(1)}Ge_{1.0(1)}P_{14}$ (**crenel** model)

| function | ADP parameter | P2 | P3 | P4 | P5 |
|---|---|---|---|---|---|
| sin1 | $U_{11}$ | 0.0045(9) | 0.0040(10) | -0.0020(10) | 0 |
| | $U_{22}$ | -0.0060(8) | 0 | 0 | 0 |
| | $U_{33}$ | 0.0053(9) | 0.0035(10) | -0.0035(10) | -0.0020(8) |
| | $U_{12}$ | 0 | 0 | 0 | 0 |
| | $U_{13}$ | 0.0039(8) | -0.0043(8) | 0.0034(8) | -0.0023(8) |
| | $U_{23}$ | 0 | 0 | 0 | 0 |
| cos1 | $U_{11}$ | 0 | 0 | 0 | 0 |
| | $U_{22}$ | 0 | 0 | 0 | 0 |
| | $U_{33}$ | 0 | 0 | 0 | 0 |
| | $U_{12}$ | 0 | -0.0020(7) | 0 | 0 |
| | $U_{13}$ | 0 | 0 | 0 | 0 |
| | $U_{23}$ | 0 | 0.0013(7) | 0 | 0 |

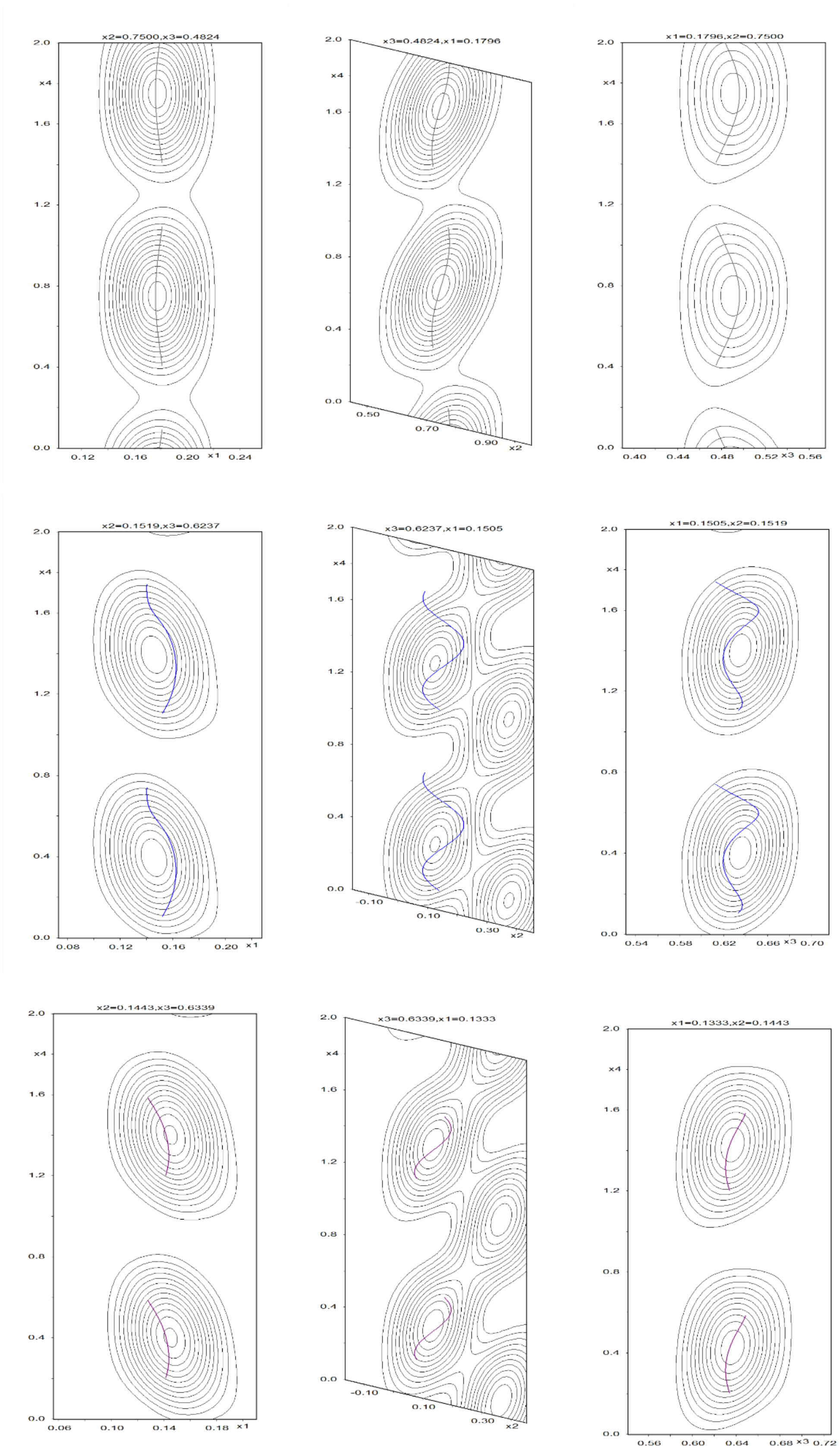


**Figure S2.** De-Wolff sections of $Ag_{1.7(1)}Ge_{1.0(1)}P_{14}$ (**crenel** model). First row: Fourier map (*x*1-*x*4, *x*2-*x*4 and *x*3-*x*4) sections around the Ag1 position; Second row: Fourier map (*x*1-*x*4, *x*2-*x*4 and *x*3-*x*4) sections around the P1 position, Fourier map (*x*1-*x*4, *x*2-*x*4 and *x*3-*x*4) sections around the Ge1 position.

## 3.3. Using the **mirror plane** model

**Table S12.** Occupancies and modulation functions of the refinement of $Ag_{1.7(1)}Ge_{1.0(1)}P_{14}$ (**mirror plane** model)

| atoms | site occupancy | ADP type | occupational modulation | positional modulation | ADP modulation |
|---|---|---|---|---|---|
| Ag1 | 0.2796 | aniso | 2 harmonics | 1 harmonics | - |
| Ge1 / P1 | 0.35(3)/ 0.706(8) | iso | 1 harmonics | 2 harmonics | - |
| P2 | 0.5 | aniso | - | 1 harmonics | 1 harmonics |
| P3 | 0.5 | aniso | - | 1 harmonics | 1 harmonics |
| P4 | 0.5 | aniso | - | 1 harmonics | 1 harmonics |
| P5 | 0.5 | aniso | - | 1 harmonics | 1 harmonics |

**Table S13.** Coefficients of the occupational modulation functions of the refinement of $Ag_{1.7(1)}Ge_{1.0(1)}P_{14}$ (**mirror plane** model)

| function | Ag1 | Ge1 | P1 |
|---|---|---|---|
| sin1 | -0.484(4) | 0.399(4) | -0.3884 |
| cos1 | 0 | 0 | 0 |
| sin2 | 0 | - | - |
| cos2 | 0.35(3) | - | - |

**Table S14.** Wyckoff positions, atomic coordinates, s.o.f. and anisotropic displacement parameters of $Ag_{1.7(1)}Ge_{1.0(1)}P_{14}$ (**mirror plane** model)

| Atom | Wyck. | *x* | *y* | *z* | s.o.f. | $U_{eq}/U_{iso}$ | $U_{11}$ | $U_{22}$ | $U_{33}$ | $U_{12}$ = $U_{23}$ | $U_{13}$ |
|---|---|---|---|---|---|---|---|---|---|---|---|
| Ag1 | 4*c* | 0.1798(3) | $\frac{3}{4}$ | 0.4821(5) | 0.2796 | 0.0225(4) | 0.0198(6) | 0.0303(7) | 0.0174(9) | 0 | 0.0034(5) |
| Ge1/P1 | 4*c* | 0.14399(16) | $\frac{1}{4}$ | 0.63686(19) | 0.147(4)/ 0.353(4) | 0.0151(7) | - | - | - | - | - |
| P2 | 4*c* | 0.32587(18) | $\frac{3}{4}$ | 0.3426(2) | 0.5 | 0.0133(7) | 0.0171(13) | 0.0066(11) | 0.0161(12) | 0 | 0.0052(9) |
| P3 | 4*c* | 0.02967(19) | $\frac{3}{4}$ | 0.3385(2) | 0.5 | 0.0141(7) | 0.0165(12) | 0.0104(11) | 0.0155(12) | 0 | -0.0052(9) |
| P4 | 4*c* | 0.06348(19) | $\frac{1}{4}$ | 0.2078(2) | 0.5 | 0.0151(7) | 0.0166(12) | 0.0107(11) | 0.0180(12) | 0 | -0.0034(10) |
| P5 | 4*c* | 0.42260(19) | $\frac{1}{4}$ | 0.4147(2) | 0.5 | 0.0135(7) | 0.0202(12) | 0.0074(10) | 0.0130(11) | 0 | 0.0035(10) |

**Table S15.** Coefficients of the positional modulation functions of the refinement of $Ag_{1.7(1)}Ge_{1.0(1)}P_{14}$ (**mirror plane** model)

| function | axis | Ag1 | Ge1/P1 | P2 | P3 | P4 | P5 |
|---|---|---|---|---|---|---|---|
| sin1 | *x* | 0.0036(6) | 0.0061(2) | -0.0038(3) | -0.0013(3) | -0.0062(3) | -0.0051(3) |
| | *y* | 0 | 0 | 0 | 0 | 0 | 0 |
| | *z* | -0.0149(11) | -0.0063(3) | -0.0061(3) | 0.0057(3) | 0.0082(3) | -0.0091(3) |
| cos1 | *x* | 0 | 0 | 0 | 0 | 0 | 0 |
| | *y* | 0.0297(7) | 0.1220(15) | -0.0217(11) | -0.0250(11) | 0.0042(11) | 0.0059(11) |
| | *z* | 0 | 0 | 0 | 0 | 0 | 0 |
| sin2 | *x* | - | 0 | - | - | - | - |
| | *y* | - | 0.0806(18) | - | - | - | - |
| | *z* | - | 0 | - | - | - | - |
| cos2 | *x* | - | 0 | - | - | - | - |
| | *y* | - | 0 | - | - | - | - |
| | *z* | - | 0 | - | - | - | - |

**Table S16.** Coefficients of the ADP modulation functions of the refinement of $Ag_{1.7(1)}Ge_{1.0(1)}P_{14}$ (**mirror plane** model)

| function | ADP parameter | P2 | P3 | P4 | P5 |
|---|---|---|---|---|---|
| sin1 | $U_{11}$ | 0.0030(18) | 0.0039(19) | -0.0019(19) | -0.001(2) |
| | $U_{22}$ | 0 | 0 | 0 | 0 |
| | $U_{33}$ | 0.0034(18) | 0.004(2) | -0.0039(19) | -0.0013(17) |
| | $U_{12}$ | 0 | 0 | 0 | 0 |
| | $U_{13}$ | 0.0040(16) | - 0.0032(15) | 0.0041(15) | -0.0007(15) |
| | $U_{23}$ | 0 | 0 | 0 | 0 |
| cos1 | $U_{11}$ | 0 | 0 | 0 | 0 |
| | $U_{22}$ | 0 | 0 | 0 | 0 |
| | $U_{33}$ | 0 | 0 | 0 | 0 |
| | $U_{12}$ | 0 | -0.0024(6) | 0 | 0 |
| | $U_{13}$ | 0 | 0 | 0 | 0 |
| | $U_{23}$ | 0 | 0 | 0.0016(16) | 0 |

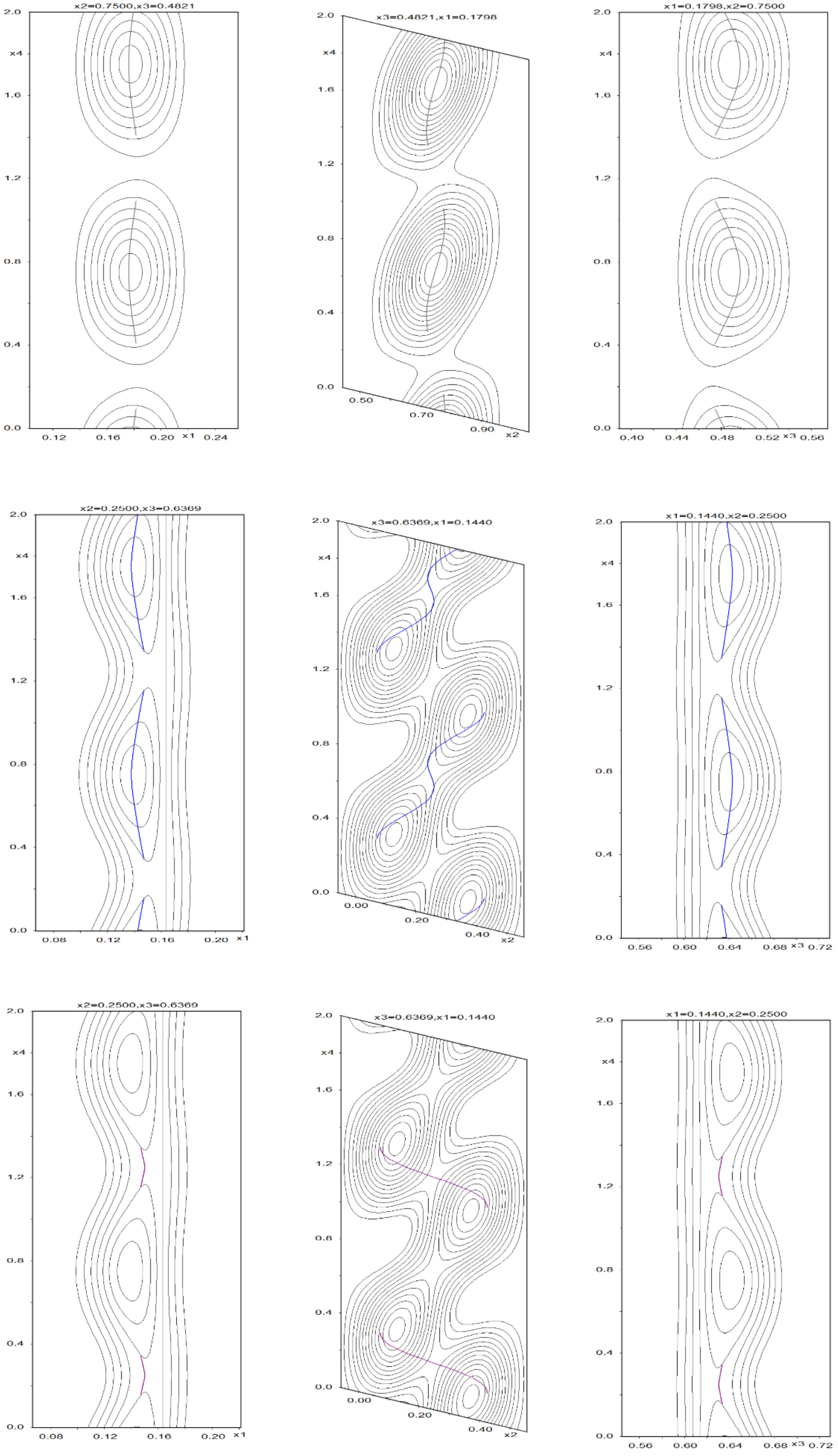


**Figure S3.** De-Wolff sections of $Ag_{1.7(1)}Ge_{1.0(1)}P_{14}$ (**mirror plane** model). First row: Fourier map (*x*1-*x*4, *x*2-*x*4 and *x*3-*x*4) sections around the Ag1 position; Second row: Fourier map (*x*1-*x*4, *x*2-*x*4 and *x*3-*x*4) sections around the P1 position, Fourier map (*x*1-*x*4, *x*2-*x*4 and *x*3-*x*4) sections around the Ge1 position.

## 4. Crystal structure parameters and atomic displacements of $Ag_{1.4}SnP_{14}$

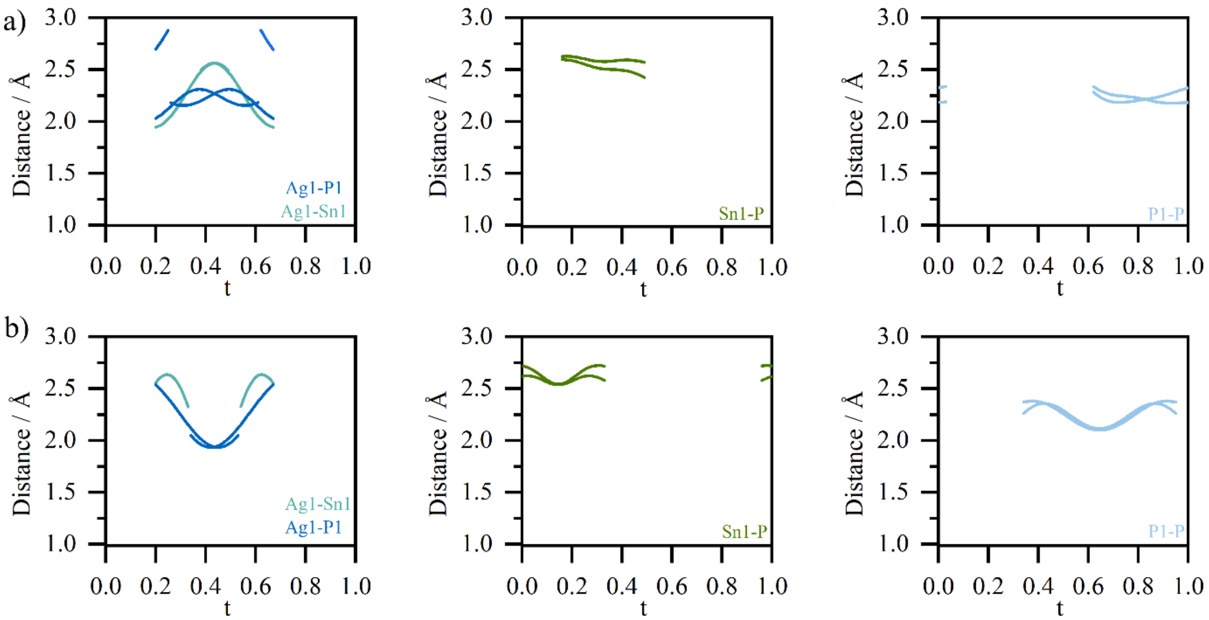


**Figure S4.** a) t-plots of the interatomic distances in $Ag_{1.4(1)}Sn_{1.0(1)}P_{14}$ : Ag1-M2(P1/Sn1) (left), M2-P (Sn1-P (middle), P1-P (right)) using the "split atom model". b) t-plots of the interatomic distances Ag1-M2(P1/Sn1) (left), M2-P (Sn1-P (middle), P1-P (right)) using the "mirror plane model".

### 4.1. Using the **split atom** model

**Table S17.** Occupancies and modulation functions of the refinement of $Ag_{1.4(1)}Sn_{1.0(1)}P_{14}$ (**split atom** model)

| atoms | site occupancy | ADP type | occupational modulation | positional modulation | ADP modulation |
|---|---|---|---|---|---|
| Ag1 | 0.4703 | aniso | 1 harmonics | 1 harmonics | - |
| Sn1/P1 | 0.1651(19) / 0.3349 | aniso | 1 harmonics | 2 harmonics | - |
| P2 | 0.5 | aniso | - | 1 harmonics | 1 harmonics |
| P3 | 0.5 | aniso | - | 1 harmonics | 1 harmonics |
| P4 | 0.5 | aniso | - | 1 harmonics | 1 harmonics |
| P5 | 0.5 | aniso | - | 1 harmonics | 1 harmonics |

**Table S18.** Wyckoff positions, atomic coordinates, s.o.f. and anisotropic displacement parameters of $Ag_{1.4(1)}Sn_{1.0(1)}P_{14}$ (**split atom** model)

| Atom | Wyck. | $x$ | $y$ | $z$ | s.o.f. | $U_{eq}/U_{iso}$ | $U_{11}$ | $U_{22}$ | $U_{33}$ | $U_{12}$ | $U_{13}$ | $U_{23}$ |
|---|---|---|---|---|---|---|---|---|---|---|---|---|
| Ag1 | 4$c$ | 0.1800(4) | $\frac{3}{4}$ | 0.4797(5) | 0.2351 | 0.0389(4) | 0.0268(5) | 0.0552(8) | 0.0348(7) | 0 | 0.0040(5) | 0 |
| Sn1 | 8$d$ | 0.1603(6) | 0.2857(14) | 0.6214(7) | 0.1651(19) | 0.0292(6) | 0.0405(10) | 0.0187(12) | 0.0285(8) | -0.0061(9) | -0.0065(7) | 0.0025(8) |
| P1 | 8$d$ | 0.1389(6) | 0.124(6) | 0.6401(6) | 0.3349(19) | 0.0292(6) | 0.0405(10) | 0.0187(12) | 0.0285(8) | -0.0061(9) | -0.0065(7) | 0.0025(8) |
| P2 | 4$c$ | 0.32630(14) | $\frac{3}{4}$ | 0.34370(16) | 0.5 | 0.0234(5) | 0.0266(8) | 0.0177(9) | 0.0261(8) | 0 | 0.0042(7) | 0 |
| P3 | 4$c$ | 0.03087(13) | $\frac{3}{4}$ | 0.33981(15) | 0.5 | 0.0228(5) | 0.0283(9) | 0.0166(8) | 0.0234(8) | 0 | -0.0043(7) | 0 |
| P4 | 4$c$ | 0.06393(13) | $\frac{1}{4}$ | 0.20932(16) | 0.5 | 0.0221(5) | 0.0230(8) | 0.0139(9) | 0.0294(8) | 0 | -0.0018(7) | 0 |
| P5 | 4$c$ | 0.42361(13) | $\frac{1}{4}$ | 0.41465(16) | 0.5 | 0.0224(5) | 0.0321(9) | 0.0157(9) | 0.0194(8) | 0 | 0.0031(7) | 0 |

**Table S19.** Coefficients of the occupational modulation functions of the refinement of $Ag_{1.4(1)}Sn_{1.0(1)}P_{14}$ (**split atom** model)

| function | Ag1 | Sn1 | P1 |
|---|---|---|---|
| sin1 | -0.518(3) | 0.225(5) | -0.470(10) |
| cos1 | 0 | -0.64(2) | 0.49(2) |

**Table S20.** Coefficients of the positional modulation functions of the refinement of $Ag_{1.4(1)}Sn_{1.0(1)}P_{14}$ (**split atom** model)

| function | axis | Ag1 | Sn1/P1 | P2 | P3 | P4 | P5 |
|---|---|---|---|---|---|---|---|
| sin1 | $x$ | 0.0029(6) | -0.0011(10) | -0.0022(3) | -0.0011(3) | -0.0061(3) | -0.0041(3) |
| | $y$ | 0 | 0.030(2) | 0 | 0 | 0 | 0 |
| | $z$ | -0.0160(7) | -0.0027(10) | -0.0069(3) | 0.0030(3) | 0.0063(3) | -0.0082(3) |
| cos1 | $x$ | 0 | -0.0033(2) | 0 | 0 | 0 | 0 |
| | $y$ | 0.0292(3) | 0.086(3) | 0.0194(10) | 0.0184(10) | -0.0046(9) | -0.0054(10) |
| | $z$ | 0 | 0.0026(3) | 0 | 0 | 0 | 0 |
| sin2 | $x$ | - | -0.0019(3) | - | - | - | - |
| | $y$ | - | -0.073(3) | - | - | - | - |
| | $z$ | - | 0.0015(4) | - | - | - | - |
| cos2 | $x$ | - | -0.0021(6) | - | - | - | - |
| | $y$ | - | -0.0753(14) | - | - | - | - |
| | $z$ | - | 0.0010(7) | - | - | - | - |

**Table S21.** Coefficients of the ADP modulation functions of the refinement of $Ag_{1.4(1)}Sn_{1.0(1)}P_{14}$ (**split atom** model)

| function | ADP parameter | P2 | P3 | P4 | P5 |
|---|---|---|---|---|---|
| sin1 | $U_{11}$ | 0.0088(15) | 0.0109(18) | -0.0004(16) | -0.0003(18) |
| | $U_{22}$ | -0.0026(14) | -0.0055(15) | -0.0121(13) | -0.0057(14) |
| | $U_{33}$ | 0.0089(16) | 0.0091(18) | -0.0017(19) | -0.0023(15) |
| | $U_{12}$ | 0 | 0 | 0 | 0 |
| | $U_{13}$ | 0.0042(18) | -0.0032(16) | 0.0000(16) | 0.0002(15) |
| | $U_{23}$ | 0 | 0 | 0 | 0 |
| cos1 | $U_{11}$ | 0 | 0 | 0 | 0 |
| | $U_{22}$ | 0 | 0 | 0 | 0 |
| | $U_{33}$ | 0 | 0 | 0 | 0 |
| | $U_{12}$ | -0.0011(15) | -0.0010(14) | -0.0033(12) | -0.0061(15) |
| | $U_{13}$ | 0 | 0 | 0 | 0 |
| | $U_{23}$ | -0.0073(15) | 0.0052(15) | 0.0056(15) | -0.0069(15) |

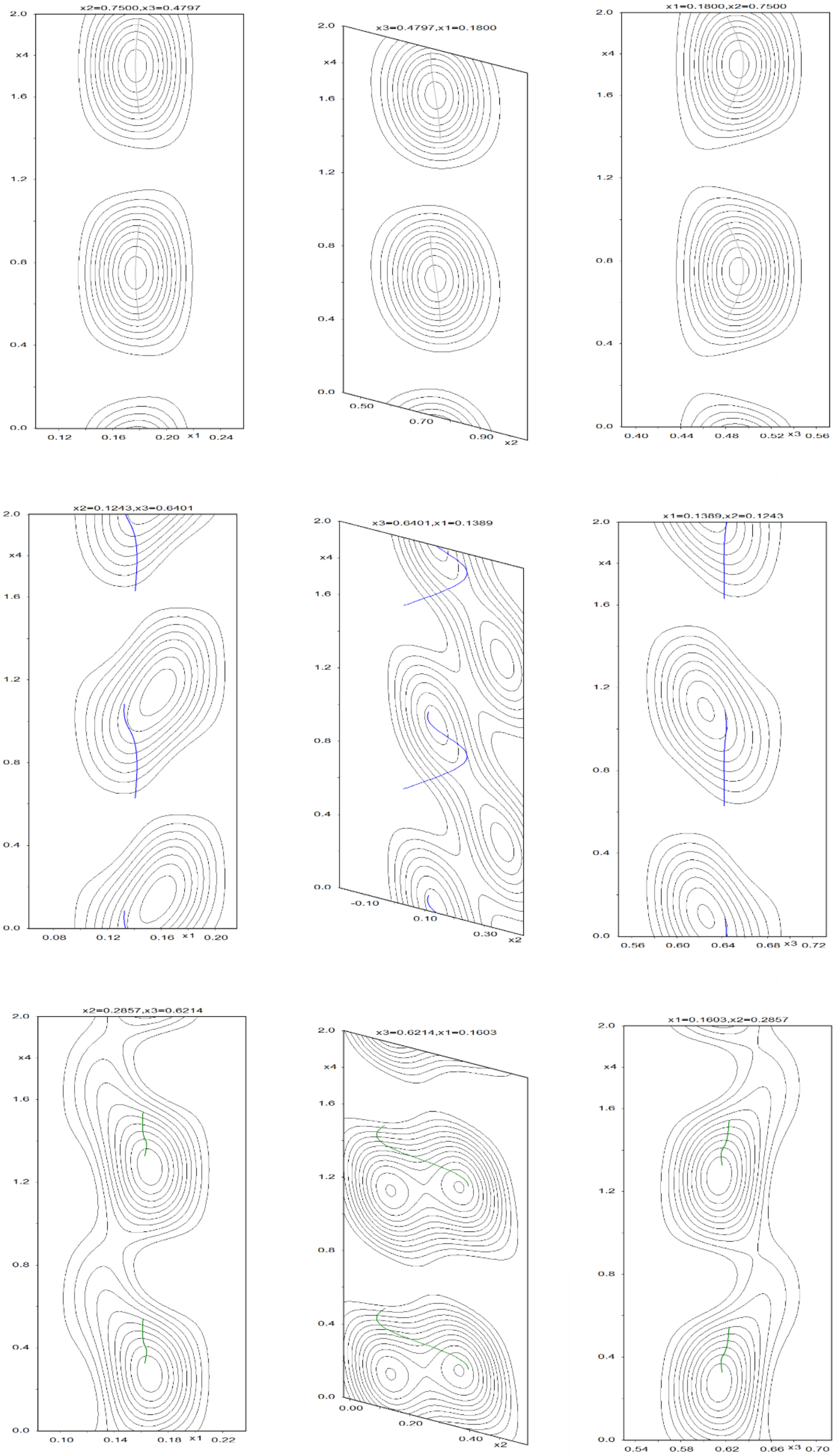


**Figure S5.** De-Wolff sections of $Ag_{1.4(1)}Sn_{1.0(1)}P_{14}$ (**split atom** model). First row: Fourier map (*x*1-*x*4, *x*2-*x*4 and *x*3-*x*4) sections around the Ag1 position; Second row: Fourier map (*x*1-*x*4, *x*2-*x*4 and *x*3-*x*4) sections around the P1 position, Fourier map (*x*1-*x*4, *x*2-*x*4 and *x*3-*x*4) sections around the Sn1 position.

## 4.2. Using the **mirror plane** model

**Table S22.** Occupancies and modulation functions of the refinement of $Ag_{1.4}SnP_{14}$ (**mirror plane** model)

| atoms | site occupancy | ADP type | occupational modulation | positional modulation | ADP modulation |
|---|---|---|---|---|---|
| Ag1 | 0.466(3) | aniso | 1 harmonics | 1 harmonics | - |
| Sn1/P1 | 0.304(5)/ 0.695 | iso | 1 harmonics | 2 harmonics | - |
| P2 | 0.5 | aniso | - | 1 harmonics | 1 harmonics |
| P3 | 0.5 | aniso | - | 1 harmonics | 1 harmonics |
| P4 | 0.5 | aniso | - | 1 harmonics | 1 harmonics |
| P5 | 0.5 | aniso | - | 1 harmonics | 1 harmonics |

**Table S23.** Wyckoff positions, atomic coordinates, s.o.f. and anisotropic displacement parameters of $Ag_{1.4}SnP_{14}$ (**mirror plane** model)

| Atom | Wyck. | *x* | *y* | *z* | s.o.f. | $U_{eq}/U_{iso}$ | $U_{11}$ | $U_{22}$ | $U_{33}$ | $U_{12}$ | $U_{13}$ | $U_{23}$ |
|---|---|---|---|---|---|---|---|---|---|---|---|---|
| Ag1 | 4*c* | 0.1808(5) | $\frac{3}{4}$ | 0.4805(6) | 0.2330(17) | 0.0394(6) | 0.0279(7) | 0.0549(12) | 0.0355(10) | 0 | 0.0043(7) | 0 |
| Sn1 | 8*d* | 0.1617(8) | $\frac{1}{4}$ | 0.6203(10) | 0.152(3) | 0.0318(9) | - | - | - | - | - | - |
| P1 | 8*d* | 0.1333(10) | $\frac{1}{4}$ | 0.6431(10) | 0.348(3) | 0.0154(11 | - | - | - | - | - | - |
| P2 | 4*c* | 0.3262(2) | $\frac{3}{4}$ | 0.3437(2) | 0.5 | 0.0242(7) | 0.0275(12) | 0.0184(12) | 0.0267(12) | 0 | 0.0038(10) | 0 |
| P3 | 4*c* | 0.03098(19) | $\frac{3}{4}$ | 0.3397(2) | 0.5 | 0.0231(7) | 0.0303(13) | 0.0149(12) | 0.0240(11) | 0 | -0.0047(10) | 0 |
| P4 | 4*c* | 0.06385(19) | $\frac{1}{4}$ | 0.2095(2) | 0.5 | 0.0241(7) | 0.0258(12) | 0.0155(13) | 0.0311(11) | 0 | -0.0028(10) | 0 |
| P5 | 4*c* | 0.42372(18) | $\frac{1}{4}$ | 0.4146(2) | 0.5 | 0.0231(7) | 0.0329(13) | 0.0157(12) | 0.0208(11) | 0 | 0.0033(10) | 0 |

**Table S24.** Coefficients of the occupational modulation functions of the refinement of $Ag_{1.4}SnP_{14}$ (**mirror plane** model)

| function | Ag1 | Sn1 | P1 |
|---|---|---|---|
| sin1 | -0.507(5) | 0.563(8) | -0.563(8) |
| cos1 | 0 | 0 | 0 |

**Table S25.** Coefficients of the positional modulation functions of the refinement of $Ag_{1.4}SnP_{14}$ (**mirror plane** model)

| function | axis | Ag1 | Sn1 | P1 | P2 | P3 | P4 | P5 |
|---|---|---|---|---|---|---|---|---|
| sin1 | *x* | 0.0041(8) | 0.0080(11) | -0.0111(18) | -0.0027(4) | -0.0010(4) | -0.0052(4) | -0.0045(4) |
| | *y* | 0 | 0 | 0 | 0 | 0 | 0 | 0 |
| | *z* | -0.0144(9) | -0.0083(13) | 0.0040(19) | -0.0078(5) | 0.0039(4) | 0.0055(5) | -0.0090(4) |
| cos1 | *x* | 0 | 0 | 0 | 0 | 0 | 0 | 0 |
| | *y* | -0.0202(11) | -0.101(7) | -0.144(3) | 0.0218(14) | 0.0261(14) | 0.0060(15) | -0.0053(15) |
| | *z* | 0 | 0 | 0 | 0 | 0 | 0 | 0 |
| sin2 | *x* | - | 0 | 0 | - | - | - | - |
| | *y* | - | 0.232(5) | 0.002(4) | - | - | - | - |
| | *z* | - | 0 | 0 | - | - | - | - |
| cos2 | *x* | - | 0.0100(7) | 0.0119(11) | - | - | - | - |
| | *y* | - | 0 | 0 | - | - | - | - |
| | *z* | - | -0.0072(9) | -0.0116(14) | - | - | - | - |

**Table S26.** Coefficients of the ADP modulation functions of the refinement of $Ag_{1.4}SnP_{14}$ (**mirror plane** model)

| function | ADP parameter | P2 | P3 | P4 | P5 |
|---|---|---|---|---|---|
| sin1 | $U_{11}$ | 0.006(2) | 0.009(2) | 0.002(2) | -0.002(2) |
| | $U_{22}$ | -0.002(2) | 0.000(2) | -0.002(2) | -0.001(2) |
| | $U_{33}$ | 0.004(2) | 0.009(2) | -0.002(3) | -0.002(2) |
| | $U_{12}$ | 0 | 0 | 0 | 0 |
| | $U_{13}$ | 0.008(2) | -0.001(2) | 0.002(2) | -0.004(2) |
| | $U_{23}$ | 0 | 0 | 0 | 0 |
| cos1 | $U_{11}$ | 0 | 0 | 0 | 0 |
| | $U_{22}$ | 0 | 0 | 0 | 0 |
| | $U_{33}$ | 0 | 0 | 0 | 0 |
| | $U_{12}$ | 0.005(2) | 0.001(2) | -0.0127(19) | -0.002(2) |
| | $U_{13}$ | 0 | 0 | 0 | 0 |
| | $U_{23}$ | -0.003(2) | 0.002(2) | 0.010(2) | -0.005(2) |

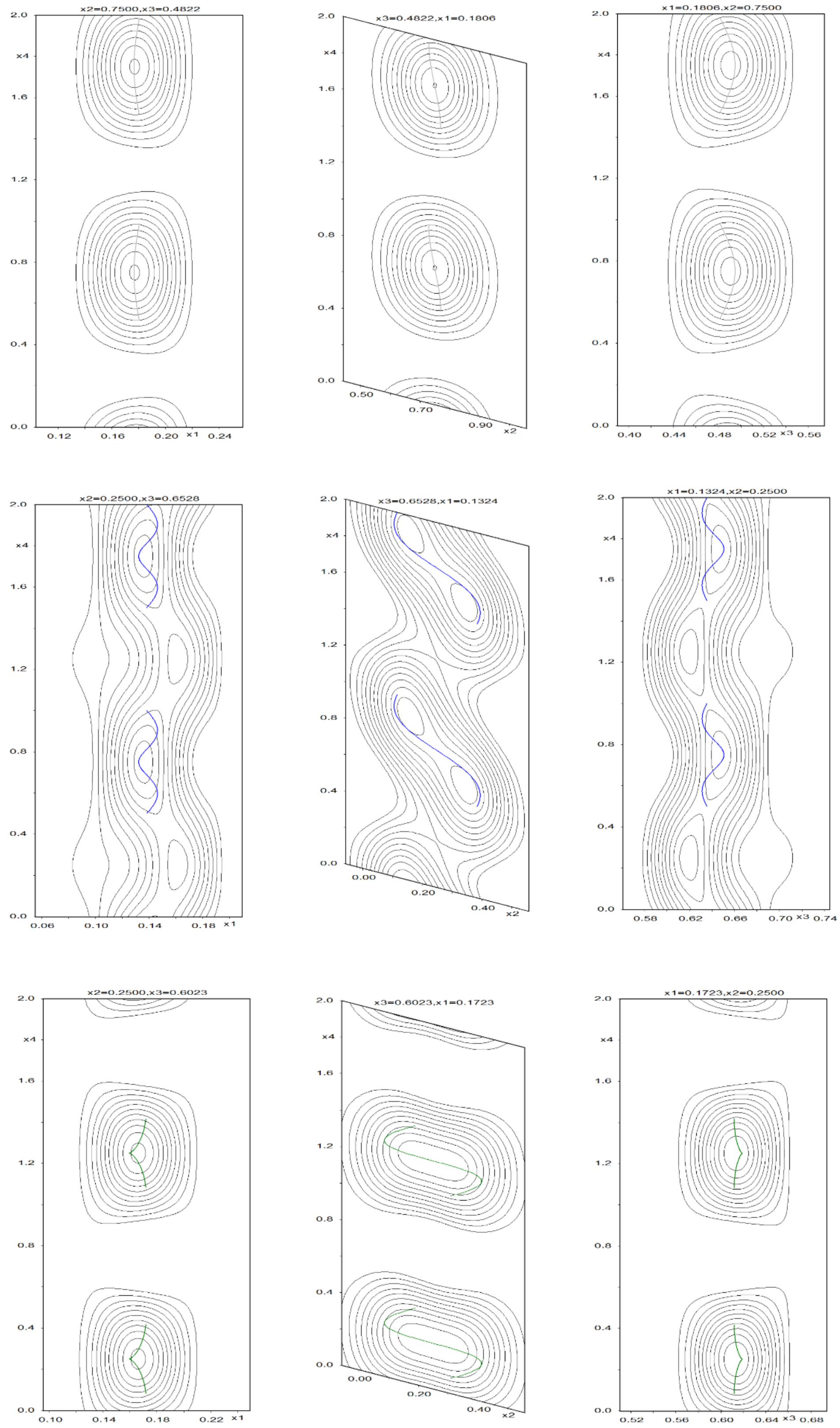


**Figure S6.** De-Wolff sections of $Ag_{1.4(1)}Sn_{1.0(1)}P_{14}$ (**split atom** model). First row: Fourier map (*x*1-*x*4, *x*2-*x*4 and *x*3-*x*4) sections around the Ag1 position; Second row: Fourier map (*x*1-*x*4, *x*2-*x*4 and *x*3-*x*4) sections around the P1 position, Fourier map (*x*1-*x*4, *x*2-*x*4 and *x*3-*x*4) sections around the Sn1 position.

## **5.** Le Bail fit of the powder diffractograms

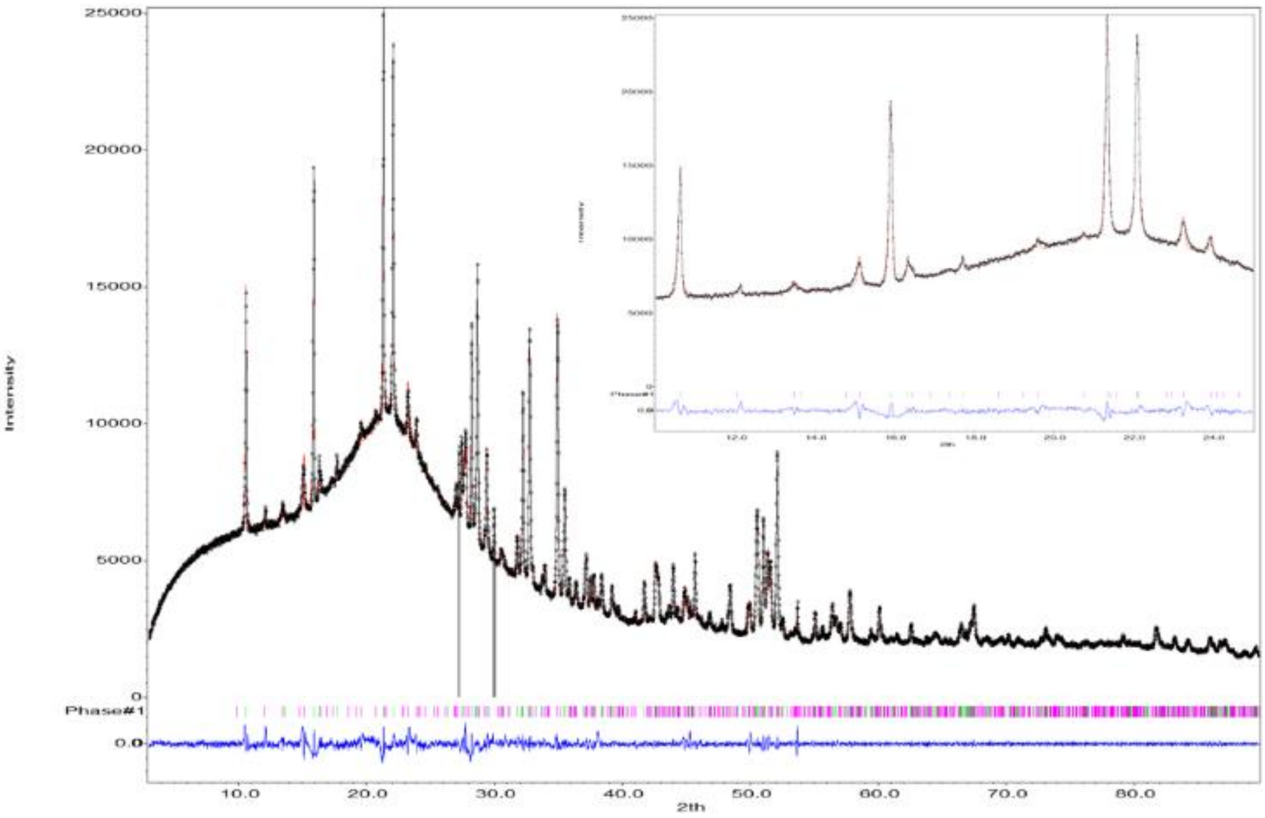


**Figure S7.** Le Bail fit on powder diffraction pattern of $Ag_{2.076}Ge_{1.6}P_{18.4}$ measured between 5-95°2θ; the inset shows the diffractogram in the range between 10-25°2θ. The green lines mark the positions calculated from crystallographic information file of the main reflections, the pink lines mark the positions of the first and second order satellite reflections. The regions containing reflections of an unknown side phase are excluded from the fit and are marked in grey.

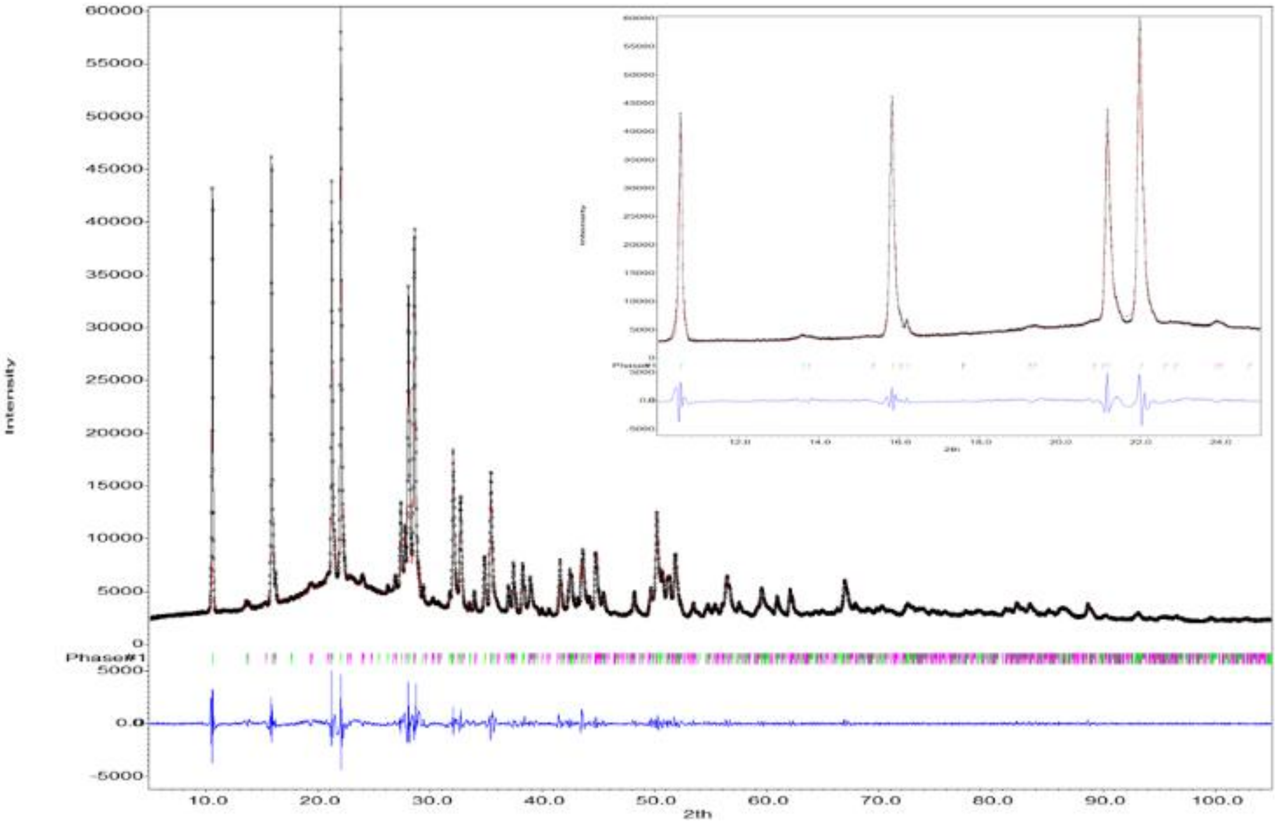


**Figure S8.** Le Bail fit on powder diffraction pattern of $Ag_{2.076}Sn_{1.6}P_{18.4}$ measured between 5-105°2θ, the green lines mark the positions calculated from crystallographic information file of the main reflections, the pink lines mark the positions of the first and second order satellite reflections; the inset shows the diffractogram in the range of 10-25°2θ.

## 6. Resistivity measurements

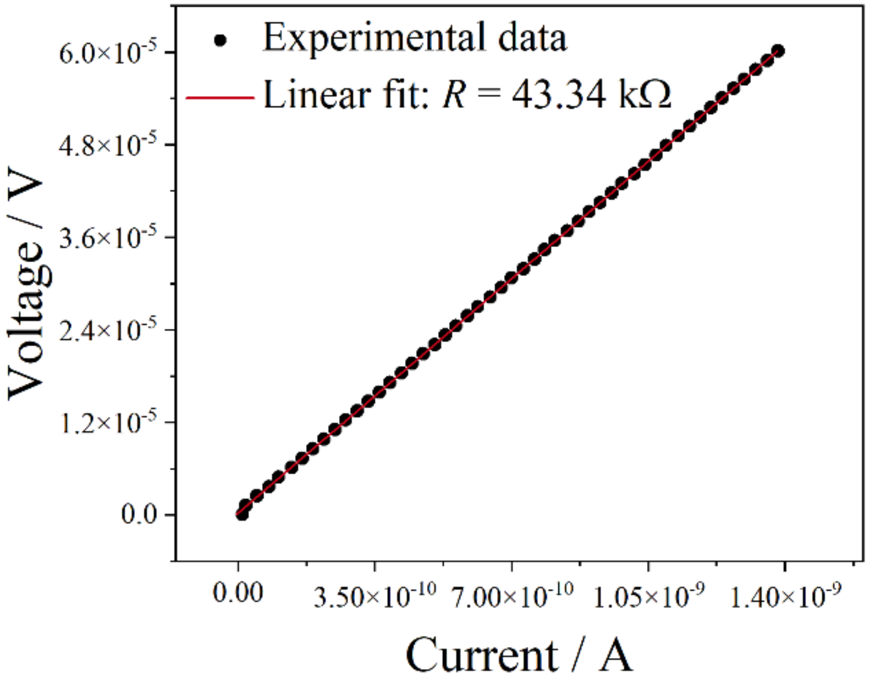


**Figure S9**. I-V curve with a linear fit (red) to determine the resistance of $Ag_{1.7(1)}Ge_{1.0(1)}P_{14}$.

In order to calculate the resistivity/ conductivity, the sample dimensions were determined to be 0.6 mm in length and of 0.03 mm in diameter, assuming a cylindrical sample geometry.
